# Whole Genome Methylation Analysis Reveals Role of DNA Methylation in Cow’s Ileal and Ileal Lymph Node Responses to *Mycobacterium avium* subsp. *paratuberculosis* Infection

**DOI:** 10.3389/fgene.2021.797490

**Published:** 2021-12-21

**Authors:** Eveline M. Ibeagha-Awemu, Nathalie Bissonnette, Suraj Bhattarai, Mengqi Wang, Pier-Luc Dudemaine, Stephanie McKay, Xin Zhao

**Affiliations:** ^1^ Sherbrooke Research and Development Centre, Agriculture and Agri-Food Canada, Sherbrooke, QC, Canada; ^2^ Department of Animal and Veterinary Sciences, University of Vermont, Burlington, VT, United States; ^3^ Department of Animal Science, McGill University, Ste-Anne-Be-Bellevue, QC, Canada

**Keywords:** DNA methylation, Johne’s disease, *Mycobacterium avim* subsp. *paratuberculosis*, dairy cow, ileum and ileum lymph node, differentially methylated cytosines and differentially methylated regions

## Abstract

Johne’s Disease (JD), caused by *Mycobacterium avium subsp paratuberculosis* (MAP), is an incurable disease of ruminants and other animal species and is characterized by an imbalance of gut immunity. The role of MAP infection on the epigenetic modeling of gut immunity during the progression of JD is still unknown. This study investigated the DNA methylation patterns in ileal (IL) and ileal lymph node (ILLN) tissues from cows diagnosed with persistent subclinical MAP infection over a one to 4 years period. DNA samples from IL and ILLN tissues from cows negative (MAPneg) (*n* = 3) or positive for MAP infection (MAPinf) (*n* = 4) were subjected to whole genome bisulfite sequencing. A total of 11,263 and 62,459 differentially methylated cytosines (DMCs), and 1259 and 8086 differentially methylated regions (DMRs) (FDR<0.1) were found between MAPinf and MAPneg IL and ILLN tissues, respectively. The DMRs were found on 394 genes (denoted DMR genes) in the IL and on 1305 genes in the ILLN. DMR genes with hypermethylated promoters/5′UTR [3 (IL) and 88 (ILLN)] or hypomethylated promoters/5′UTR [10 (IL) and 25 (ILLN)] and having multiple functions including response to stimulus/immune response (*BLK, BTC, CCL21, AVPR1A, CHRNG, GABRA4, TDGF1*), cellular processes (*H2AC20, TEX101, GLA, NCKAP5L, RBM27, SLC18A1, H2AC20BARHL2, NLGN3, SUV39H1, GABRA4, PPA1, UBE2D2*) and metabolic processes (*GSTO2, H2AC20, SUV39H1, PPA1, UBE2D2*) are potential DNA methylation candidate genes of MAP infection. The ILLN DMR genes were enriched for more biological process (BP) gene ontology (GO) terms (*n* = 374), most of which were related to cellular processes (27.6%), biological regulation (16.6%), metabolic processes (15.4%) and response to stimulus/immune response (8.2%) compared to 75 BP GO terms (related to cellular processes, metabolic processes and transport, and system development) enriched for IL DMR genes. ILLN DMR genes were enriched for more pathways (*n* = 47) including 13 disease pathways compared with 36 enriched pathways, including 7 disease/immune pathways for IL DMR genes. In conclusion, the results show tissue specific responses to MAP infection with more epigenetic changes (DMCs and DMRs) in the ILLN than in the IL tissue, suggesting that the ILLN and immune processes were more responsive to regulation by methylation of DNA relative to IL tissue. Our data is the first to demonstrate a potential role for DNA methylation in the pathogenesis of MAP infection in dairy cattle.

## Introduction

Infection with *Mycobacterium avium* subsp*. paratuberculosis* (MAP), the causative agent of Johne’s disease (JD) of bovine and other ruminants, is one of the major threats to cattle health and the economic profitability of the dairy industry (Whittington et al., 2019). JD is a long progressive and chronic enteric disease which progresses through three phases upon MAP infection (silent, subclinical, and clinical/advanced stages) and is characterized by imbalance in gut immunity, and MAP’s ability to escape host immune surveillance, and subvert host cell responses to ensure its intracellular survival and replication (Whitlock and Buergelt, 1996; Arsenault et al., 2014; Koets et al., 2015). Similar with other mycobacterial organisms, MAP infects and persists first in macrophages and is found in lymphatic tissues before inducing pathology in the gut or elsewhere, as a secondary process. MAP acquired propensity to infect macrophages helps it to exquisitely adapt and survive in an aggressive host immune response environment. The long-term subclinical period (2–5 years) between the infection period (phase I) and the clinical period (phase III) of JD provides an opportunity for MAP to adapt its environment for its survival. It is known that, pathogenic mycobacteria, including the *Mycobacterium avium* complex pathogens, respond to host immune responses with counterattack strategies, notably to escape the maturation of phagolysosomes of the phagocytotic cells, which function to destroy them (Liu and Modlin, 2008). The involvement of epigenetic mechanisms in the regulation of MAP survival in the host has been proposed (Ariel et al., 2020), requiring further investigations. Furthermore, reported potential connection between JD and human diseases like Crohn’s disease (CD) and arthritis rheumatoid (Over et al., 2011; Mcnees et al., 2015; Timms et al., 2016; Bo et al., 2020) emphasizes the importance of controlling JD. In addition, the effect of JD on animals, the environment and the economy highlights the need for deeper understanding of the host cellular processes that are perturbed or manipulated by MAP, in order to develop robust disease control strategies.

Several recent reviews have documented the contribution of epigenetic regulation (DNA methylation, histone modifications and miRNA and lncRNA expression) to economically important livestock traits, including disease susceptibility and the immune response (Doherty et al., 2014; Ibeagha-Awemu and Zhao, 2015; Triantaphyllopoulos et al., 2016; Thompson et al., 2020; Wang and Ibeagha-Awemu, 2021). For instance, miRNAs have been found to regulate the autophagic response to eliminate intracellular *Mycobacterium* (Wang et al., 2013; Wang et al., 2020), modulate inflammatory signaling and to control pathogen survival and replication including *Mycobacterium tuberculosis* (Sharbati et al., 2011; Mehta and Liu, 2014). Moreover, altered expression of miRNAs during bacterial (mycobacteria, salmonellae and listeriae) invasion of host cells have been shown to affect immune signalling pathways including apoptosis and autophagy related pathways (Sharbati et al., 2011; Hoeke et al., 2013; Pawar et al., 2016; Zur Bruegge et al., 2017). Meanwhile, DNA methylation, especially in CpG (cytosine-phosphate-guanosine) context, has been demonstrated for several cattle disease conditions caused by various pathogens, including *Mycobacterium bovis* (Doherty et al., 2016), etc. For example, the bovine CD4^+^ T cells methylome comparison revealed 196 differentially methylated regions (DMRs) that directly contributed to differential gene expression including key inflammatory genes in CD4^+^ cells isolated from *Mycobacterium bovis*-infected cows (Doherty et al., 2016). In addition, an *in vitro* study found that DNA methylation could regulate NLRP3 promoter activity and its gene expression, thereby implicating DNA methylation in NLRP3 inflammasome activation in response to *Mycobacterium tuberculosis* infection (Wei et al., 2016). Our recent studies reported that MAP impacted the expression of epigenetics genes (Ariel et al., 2019), the lncRNA pattern of macrophage, and the first host immune cell defense encountered while invading cows (Gupta et al., 2019; Marete et al., 2021b). Moreover, comparing the transcriptome landscape of macrophages and small intestinal tissues and draining lymph nodes from JD positive cows with JD negative cows, we observed modifications that show the apparent importance of immune and metabolic pathways hijacked by MAP to subvert the host immune system (Ariel et al., 2020; Ibeagha-Awemu et al., 2021) suggesting a role of epigenetic processes in host response to MAP infection. However, the effect of MAP on the bovine epigenome, specifically the DNA methylome remains unexplored. Considering that intracellular microbial infection can lead to alterations of the host DNA methylome; we hypothesize that epigenetics regulation would be among strategies used by MAP to establish a tolerant environment in infected cows. Therefore, this study used whole genome bisulfite sequencing (WGBS) technique to test the hypothesis that DNA methylation patterns of cow small intestinal tissues are altered during subclinical MAP infection.

## Materials and Methods

### Experimental Animals

Animal selection and diagnosis of MAP status has been described in details in our recent publication (Ibeagha-Awemu et al., 2021). Briefly, animals were from companion studies (Ariel et al., 2020; Marete et al., 2021b; Marete et al., 2021c) which includes 23 commercial dairy farms (tie and free stall) positive for JD and located in the provinces of Québec and Ontario, Canada. Blood and fecal samples collected twice a year and over a period of one to 4 years were diagnosed for MAP status as described previously (Fock-Chow-Tho et al., 2017). Two different tests, Pourquier serum ELISA assay (IDEXX Laboratories, Markham, Ontario, Canada) and MAP fecal PCR were used to diagnosis for the presence of MAP. Cows positive for both serological and fecal tests were grouped as MAP-infected (MAPinf) (*n* = 4) while cows negative for both tests constituted the MAP-negative (MAPneg) or control group (*n* = 5). Mycobacterial fecal culture (Laboratoire d’épidémiosurveillance animale du Québec, Saint-Hyacinthe, Québec, Canada) was used to confirm live MAP excretion, as previously described (Arango-Sabogal et al., 2016). Animals (*n* = 9) were humanely euthanized by intra-venous administration of 5 mg detomidin and 120 ml euthansol, and ileal (IL) and ileal lymph node (ILLN) tissues without visible Peyer’s patches were collected. Ileal tissue was taken at about 35 cm before the ileocecal valve and ILLN tissue was collected proximal to this point. Following collection of tissues, a third test, f57 real time qPCR was performed to confirm the presence or absence of MAP in the tissues according to our previous study (Ibeagha-Awemu et al., 2021). MAP was identified in tissues from all cows in the MAPinf group and its absence was demonstrated in all tissues from 3 cows out of 5 in MAPneg group (Ibeagha-Awemu et al., 2021). Only the 4 cows in MAPinf group and 3 cows in MAPneg group were further used.

### Acid Fast Staining

Tissue pieces about 3–4 mm^2^, sampled for DNA methylation analysis were immediately embedded in OCT (Optimal cutting temperature compound) and stored in sealed containers at −80°C. Tissue sections (8 μm) were cut for acid fast staining at the Plateforme d’histologie et microscopie électronique, Faculté de médecine, Université de Sherbrooke.

### Library Preparation and Whole Genome Bisulfite Sequencing

Genomic DNA was isolated from IL and ILLN tissues using DNeasy Blood and Tissue Kit (Qiagen). DNA was quantified using the Quant-iT™ PicoGreen® dsDNA Assay Kit (Life Technologies) and bisulfite converted using the EZ DNA Methylation-Lightning Kit (Zymo). Libraries were generated using 200 ng DNA/sample and the NEBNext Ultra II DNA Library Prep Kit for Illumina (New England BioLabs) according to manufacturer’s protocol, including CpG methylated pUC19 and unmethylated lambda phage spike-in controls. Size selection of libraries containing the desired insert size was achieved with SPRIselect beads (Beckman Coulter). Libraries were quantified using the Kapa Illumina GA with Revised Primers-SYBR Fast universal kit (Kapa Biosystems) and average size fragment was determined using a LabChip GX (PerkinElmer) instrument.

The libraries were normalized and pooled and then denatured in 0.05 N NaOH and neutralized using HT1 buffer. The pool was loaded at 225pM on an Illumina NovaSeq S4 lane using Xp protocol as per the manufacturer’s recommendations. The run was performed for 2 × 150 cycles (paired-end mode). A phiX library was used as a control and mixed with libraries at 5% level. Base calling was performed with RTA v3. Bcl2fastq2 v2.20 software was used to demultiplex samples and to generate fastq reads. Sequencing was performed to achieve an average depth of 20× using the Illumina NovaSeq S4 system (Illumina, United States). WGBS library preparation and sequencing was performed by the Centre d’expertise et de services Génome Québec (https://cesgq.com/).

### Bioinformatics Processing of Whole Genome Bisulfite Sequencing Data

Initial quality control on raw reads to remove low quality reads (Phred score <20) and adaptor sequences was performed with TrimGalore v0.4.4 (Krueger, 2012) and CutAdapt v1.13 (Martin, 2011) programs using the default parameters. Read quality and metrics were visualised and curated using FastQC v0.11.3 (Andrews, 2010). The bovine reference genome, ARS-UCD1.2 was indexed using BSseeker2 -v2.1.7 (Guo et al., 2013). The cleaned data for each sample were then aligned to the indexed reference genome using bowtie2 aligner within BSseeker2 (Guo et al., 2013). Mapped reads were fixmated, sorted, and PCR duplicates removed with SAMtools-v1.7 (Li et al., 2009). The methylation level in each cytosine was then determined using BSseeker2 using the default parameters. Methylation data output included read coverage and percentage of methylated cytosines at each genomic cytosine position. Methylation sites in the context of CpG (cytosine-phosphate-guanosine) and non CpG (i.e. CHG or CHH where H denotes C (cytosine), A [adenosine) or T (thymine)] were extracted for each sample. Methylated cytosines were analysed in genic context including promoter (2000 bp upstream of the transcription start site), 5′UTR, exon, intron, 3′UTR and downstream region (2000 bp downstream of the transcription termination site) of genes, and in CpG island (CGI) context including regions surrounding CGIs such as CGI shores and CGI shelves. CGI shores included the 2 kb region upstream of the CpG island (CGI shore left) and 2 kb region downstream of the CpG island (CGI shore right). CGI shelves included the 2 kb region upstream of the CpG shore left (CpG shelf left) and 2 kb region downstream of the CpG shore right (CGI shelf right). Histograms of methylation sites, coverage and methylation distribution for all samples were performed with the R package methylKit -v1.12.0 (https://bioconductor.org/packages/release/bioc/html/methylKit.html) (Akalin et al., 2012). Moreover, the methylation levels of MAP-infected and negative IL or ILLN samples were merged and density plots generated according to chromosomes using the R package, Circlize (Gu et al., 2014) (https://cran.r-project.org/web/packages/circlize/index.html).

Based on CpG methylation, correlation analysis to determine the relationship between samples and principal component analysis to visualize sample clustering was also performed with methylKit. DNA methylation dynamic plots were generated with Beanplot program in R package (https://cran.r-project.org/web/packages/beanplot/) (Kampstra, 2008).

### Differentially Methylated Cytosines and Regions, and Annotation

Methylated sites were compared between MAPinf and MAPneg groups with the Radmeth function of Methpipe -v3.4.3 (Song et al., 2013). The original *p* values generated from regression with the Radmeth were adjusted for multiple testing and combining significance in the bins of 1:200:1 which gave the false discovery rate (FDR) corrected *p* value. The annotation files for the genic region and CGIs were downloaded from the UCSC genome browser by selecting ARS-UCD1.2 assembly for Cow genome. Differentially methylated cytosines (DMCs) and differentially methylated regions (DMRs) were annotated within genic regions (promoter, 5′UTR, exon, intron, 3′UTR and downstream region) and in CGI context (CGIs, CGI shores and CGI shelves) with R package annotatr -v1.8.0 (Cavalcante and Sartor, 2017). Significantly differentially methylated cytosines and DMRs were defined as having Benjaminin and Hochberg (Benjamini and Hochberg, 1995) corrected false discovery rate (FDR) < 0.1. DMRs were further filtered for length (<1 kb) and including at least 3 CpG sites.

### Gene Ontology and Pathways Analysis

Genes harboring DMRs were subjected to functional enrichment analysis using ClueGO (http://apps.cytoscape.org/apps/cluego) (Bindea et al., 2009), which is an APP in Cytoscape (Shannon et al., 2003). Gene ontology (GO) terms and Kyoto Encyclopedia of Genes and Genomes (KEGG) pathways were adjusted with Benjamini-Hochberg correction FDR (Benjamini and Hochberg, 1995) and considered significantly enriched at FDR <0.05. GO terms were grouped in to biological processes (BP), molecular functions (MF) and cellular component (CC) categories. Enriched KEGG pathways were visualized with ClueGO.

## Results

### MAP is Present in the Analyzed Tissues

**FIGURE 1:**
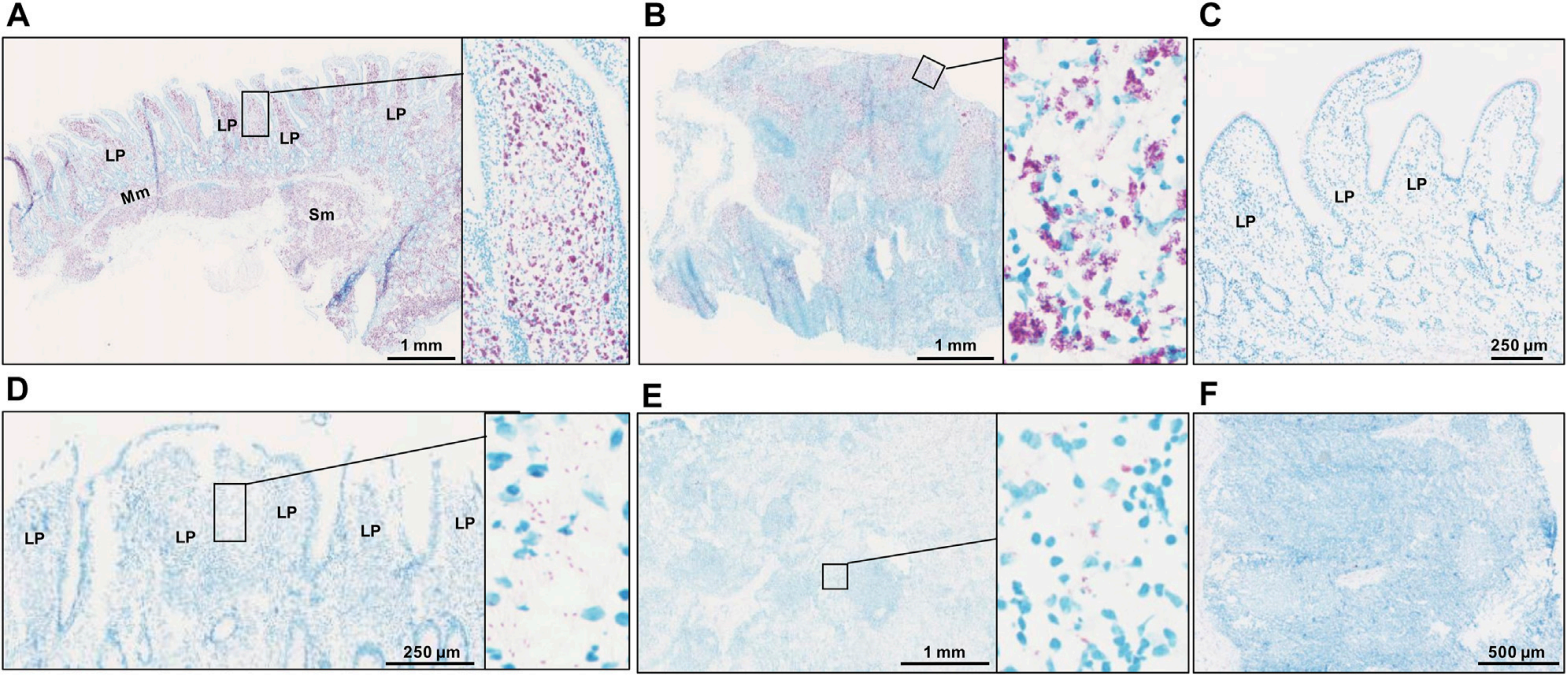
Acid-fast staining for acid-fast bacteria in ileum **(A)** and ileum lymph node **(B)** from a MAP-infected cow with high copies of MAP, showing abundant acid-fast staining (purple color). Panels **(D)** (ileum) and **(E)** (ileum lymph node) are tissues from a MAP-infected cow with fewer copies of MAP, showing very sparse acid-fast staining. No evidence of acid fast bacteria was seen in ileum **(C)** and ileum lymph node **(F)** tissues of a MAP-negative cow. LP, lamina propria; Mm, muscularis mucosa; SM, submucosa.

Following the results of f57 qPCR demonstrating the presence of MAP in the MAPinf tissues and absence in the MAPneg tissues (Ibeagha-Awemu et al., 2021), we performed acid fast staining to visualize the distribution of MAP in the analyzed tissues. The result of acid fast staining showed abundant presence of acid fast bacteria (Figures 1A,B) or sparse-acid fast staining indicative of fewer acid fast bacteria (Figures 1D,E) in IL and ILLN tissues from two MAPinf cows. Meanwhile, no evidence of acid fast bacteria indicative of the absence of MAP was seen in IL and ILLN tissues from a MAPneg cow (Figures 1C,F). This result is consistent with the copy numbers of MAP detected by the method of f57 qPCR (Ibeagha-Awemu et al., 2021).

### Mapping Statistics

**FIGURE 2:**
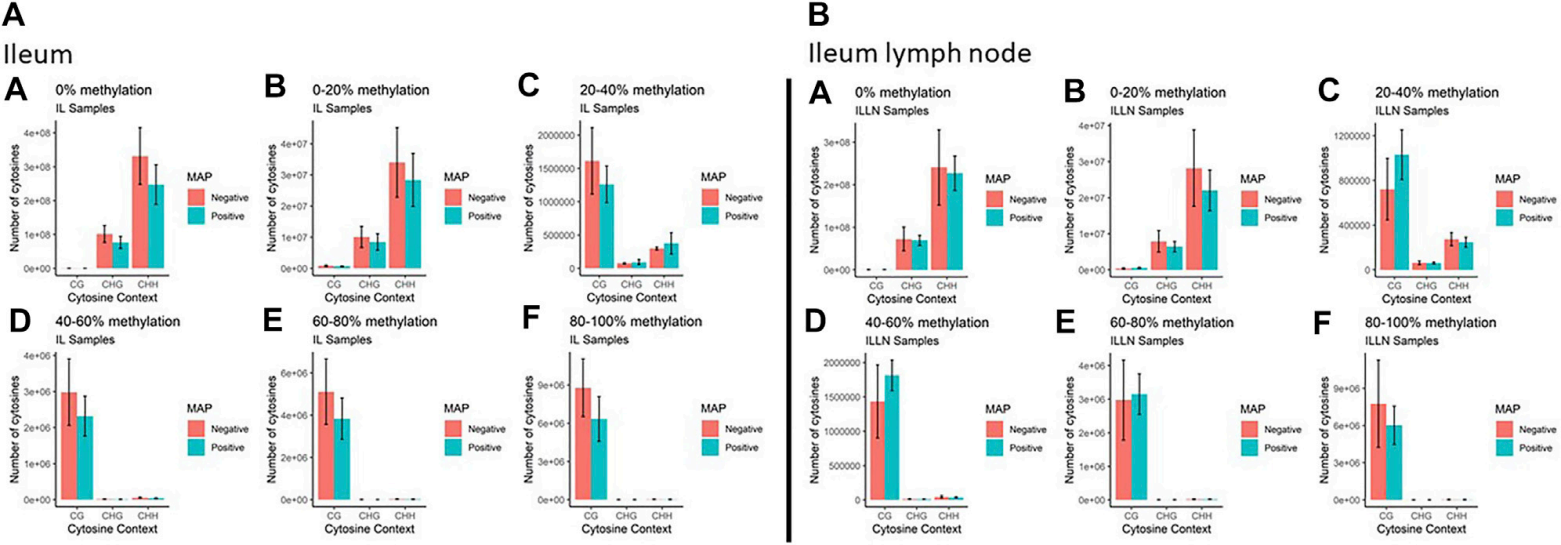
The total number of cytosines present in CpG and non-CpG (CHG and CHH, where H = A, T or C) context along with the methylation levels in MAP-infected and MAP-negative **(A)** ileum (IL) and **(B)** IL lymph node (ILLN) samples. Moving from A to F in both IL and ILLN samples, the number of cytosines and percent methylation increases in CpG context whereas it decreases in non-CpG.

Genome-wide DNA methylation sequencing was conducted on IL and ILLN tissues from four cows positive for MAP infection and three cows negative for MAP infection at a depth of 25× and read length of 150 bp. A total of 2.02 B (billion) and 1.87 B clean reads (adaptor sequences, and reads with Phred score <20 removed) were obtained for IL and ILLN samples, respectively (Supplementary Table S1). Out of these numbers, 70.39% (IL) and 71.67% (ILLN) were uniquely aligned to the bovine reference genome. Mapping rate of bases (A, T, or C) ranged from 68.93 to 71.09% for IL samples and from 69.27 to 76.79% for ILLN samples of the seven cows. The average rate of methylated cytosines in CpG (or mCG), mCHG and mCHH contexts were 67.72, 0.89, 0.90, 71.34, 0.86 and 0.87% in IL and ILLN samples, respectively (Supplementary Table S1). Examination of the percentage of methylation in the various contexts indicated that, the number of cytosines and percent methylation increased in CpG context whereas it decreased in non-CpG context (Figure 2). Since methylation rates in the mCHG and mCHH contexts were very low, subsequent analyses were based on CpG context only.

### Global Mapping of DNA Methylation Status

A total of 44.5 million (M) (MAPinf) and 38.6 M (MAPneg), and 38.8 M (MAPinf) and 28.4 M (MAPneg) CpG sites with read coverage per base ≥10× were found in IL and ILLN samples respectively. Out of these, 15.7 M (including 1,342,23 sites in genic regions) and 12.3 M (including 1,246,608 sites in genic regions) methylated sites in IL and ILLN tissues of the seven cows, respectively were used to determine significantly differential methylation between MAPinf and MAPneg cows in each tissue. Pearson correlation was performed to understand the relationship between samples. As shown in Supplementary Figure S1, samples in both tissues were highly correlated with a Pearson correlation coefficient range from 0.70 to 0.79. Global chromosomal dynamics of methylated cytosine distribution in each group and tissue are shown in Supplementary Figure S2. To gain further insight into the methylation profiles, the extent of methylation levels at different genic regions was also analysed. Bean plots show that most of the CpG sites in the 5′UTR and promoter had low levels of methylation (mean of ∼35 and ∼48% in IL or ∼47 and ∼57% in ILLN, respectively), whereas, most of the CpG sites in other genic regions had higher levels of methylation (mean of >72% in both tissues) (Supplementary Figures S3A,B). Moreover, the mean level of methylation was slightly higher in the promoters and intron regions of IL MAPinf samples as compared to IL MAPneg samples. The converse was true for ILLN samples whereby the methylation rates were slightly lower in ILLN MAPinf samples as compared to ILLN MAPneg samples (Supplementary Figures S3A,B).

### Differentially Methylated Cytosines Between MAP-Infected and MAP-Negative Samples, and Distribution Patterns

**FIGURE 3:**
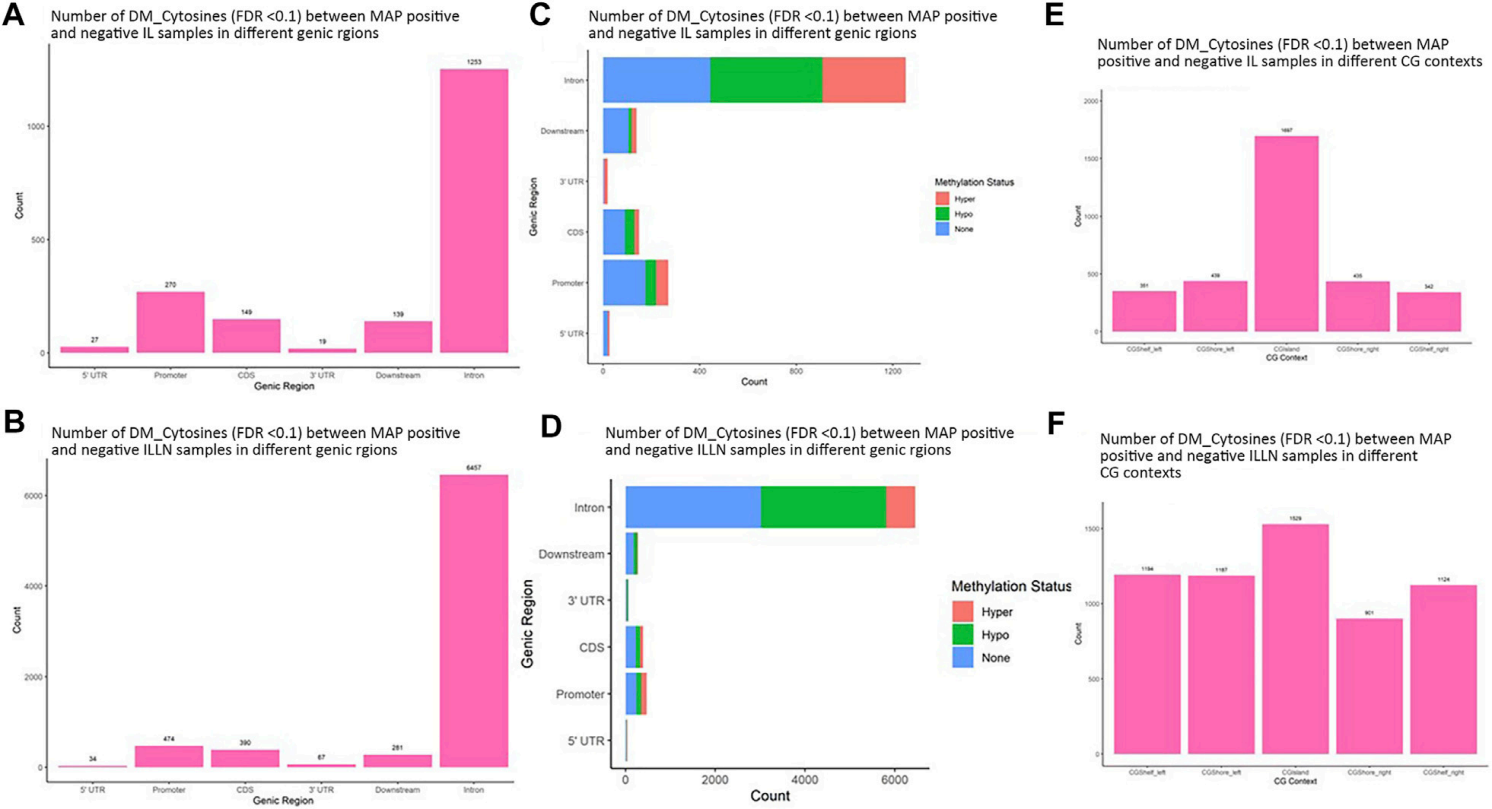
Distribution of significantly differentially methylated cytosines (DMC) (FDR<0.1) in different genic regions between MAP-infected (MAPinf) and MAP-negative (MAPneg) **(A)** ileum (IL) and **(B)** IL lymph node (ILLN) tissues, and corresponding methylation status in **(D)** IL and **(C)** ILLN. A DMC was considered hypermethylated if it had a positive methylation difference ≥25% between MAPinf and MAPneg samples. Similarly, a DMC was hypomethylated if it had a negative methylation difference ≥25% between MAPinf and MAPneg samples. A DMC methylation status of none implies that the methylation difference was less than 25% in both scenarios. Distribution of differentially methylated cytosines in CG context in **(E)** IL and **(F)** ILLN tissues between MAPinf and MAPneg samples.

A total of 11,263 and 62,459 significant DMCs (FDR<0.1) were found between MAPinf and MAPneg samples, including 1857 and 7703 in genic regions in IL and ILLN tissues, respectively (Supplementary Tables S2A–D and Supplementary Figure S4). Out of this number, 297 or 508, respectively were located in the promoter and 5′UTRs while highest numbers were located in intron regions. Chromosomal distribution of DMCs between MAPinf and MAPneg cows showed higher concentrations (*n* > 500) on *Bos taurus* chromosomes (BTA) 1, 2, 3, 4, 5, 6, 7, 8, 9 and X and lowest numbers (*n* < 100) on BTA 22, 28 and 29 in the IL (Supplementary Table S2E). Similarly, more significant DMCs (>3900) were located on BTA 1, 2, 3, 4, 5, 6, 7, 8, 9 and X and least numbers on BTA 25 (*n* = 397), 28 (n = 429) and 29 (*n* = 462) in ILLN samples (Supplementary Table S2E). A total of 835 DMCs, including 124 DMCs in genic regions, were common to both IL and ILLN tissues (Supplementary Tables S2F,G). The degree of methylation was further classified into hypermethylation (methylation difference (increase) between MAPinf and MAPneg samples is 25% or more), hypomethylation (methylation difference (decrease) between MAPinf and MAPneg samples is 25% or more) or none (neither hypermethylated or hypomethylated and having methylation difference < |25%|). In both tissues, a higher number of genic region DMCs were hypomethylated as compared to hypermethylation status (Figures 3C,D).

Distribution in CGI context indicated a higher number of significant DMCs in CGIs, CGI shores and shelves in IL (1697) as compared to 1529 in ILLN (Figures 3E,F, and Supplementary Tables S3A,B). Relative to CGIs, more DMCs were found in CGI shores left and right than in CGI shelves left and right in IL, meanwhile, CGI shore left and CGI shelf left contained slightly higher numbers of DMCs than CGI shore right and CGI shelf right, respectively in ILLN (Figures 3E,F and Supplementary Tables S3A,B).

The DMCs were annotated to 503 genes in the IL, including to the promoters and or 5′UTRs of 54 genes (Supplementary Table S2B). In the IL, the most significant DMCs were found in the promoter regions of *TMEM15* (17 DMC sites, FDR from 5.89E-07 to 1.60E-04), *CDKN2C* (13 DMC sites, FDR from 2.46E-04 to 7.57E-04), CDS of *HSPA1A* (13 DMC sites, FDR = 1.49E-05 to 9.87E-04) and introns of *CD38* (2 DMC sites, FDR = 2.78E-05 to 0.0019), *CESP-1* (16 DMC site, FDR = 1.27E-04 to 0.004), *BCL2* (22 DMC sites, FDR from 1.45E-04 to 0.003), *NTRK3* (5 DMC sites, FDR from 4.65E-04 to 7.57E-04). A total of 89 genes harbored 6 to 89 DMCs, including bta-mir-2887–1 (89 DMC sites in downstream region), *HSPA1A* (48 sites in CDS), *PGAM1* (25 DMC sites in promoter), *BCL2* (22 DMC sites in intron), *TMEM15* (22 DMC sites in promoter), *CESP-1* (16 DMC sites in intron), *VPS37B* (16 DMC sites in intron), *BCL6* (15 DMCs in promoter) and *CD34* (15 DMCs in intron) (Table 1). Moreover, two genes harbored 14 (*TKTL1*) and 12 (*TOP3A*) DMC sites in their promoters, 5′UTRs and CDS.

**TABLE 1:** Genes with hypermethylated or hypomethylated DMRs in their promoters or 5′UTRs in the ileum and ileum lymph node tissues of MAP-infected cows.

Chr^a^	Start	End	MethyStatus^b^	MethDiff^c^	# CG^d^	Gene symbol and synonyms	Genic region	Function
**Ileum**								
chr1	2767388	2767399	Hyper	0.4545	3	C1H21orf62	5′ UTR	Not available
chr26	24841896	24841962	Hyper	0.2703	5	GSTO2	5′ UTR	Involved in metabolic processes
chr1	83304091	83304285	Hyper	0.2926	14	MAP6D1	Promoter	Implicated in calmodulin binding
chr4	112058821	112059126	Hyper	0.2504	10	EZH2	Promoter	Involved in B cell differentiation, DNA methylation and histone H3K27 methylationetc.
chr6	102277355	102277489	Hyper	0.2670	6	HSD17B11	Promoter	Involved in androgen catabolic process and estrogen biosynthetic process
chr7	11812988	11813064	Hyper	0.3420	7	MIR181D	Promoter	Supports mRNA binding and is involved in posttranscriptional gene silencing
chr8	60445670	60445682	Hyper	0.5972	3	CCIN	Promoter	Roles in actin filament binding and protein binding. Involved in actin cytoskeleton organization, brain development, cell differentiation and spermatogenesis
chr19	42828468	42828675	Hyper	0.4820	11	CCDC56; COA3	Promoter	Roles in protein binding; involved in mitochondrial cytochrome c oxidase assembly and positive regulation of mitochondrial translation
chr7	50789736	50789822	Hypo	−0.3279	10	UBE2D2	5′ UTR	Involved in cellular processes, ubiquitin-protein ligase and metabolic processes
chr11	88020652	88021055	Hypo	−0.3464	6	ITGB1BP1	5′ UTR	Roles in GDP-dissociation inhibitor activity; integrin binding; protein kinase binding; and is involved in Notch signaling pathway, activation of protein kinase B activityetc.
chrX	50803770	50803957	Hypo	−0.2683	10	GLA	5′ UTR	Involved in cellular processes, galactosidase and metabolic processes
chr2	120178323	120178411	Hypo	−0.2786	4	CHRNG	Promoter	Implicated in response to stimulus and signalling
chr7	38857595	38857640	Hypo	−0.4957	6	RGS14	Promoter	Roles in G-protein alpha-subunit binding, GDP-dissociation inhibitor activity, GTPase activating protein bindingetc.
chr11	29596293	29596466	Hypo	−0.3359	4	CALM2	Promoter	Roles in N-terminal myristoylation domain binding, adenylate cyclase activator activity and disordered domain specific bindingetc.
chr13	61040934	61041114	Hypo	−0.3170	5	DEFB123	Promoter	Involved in defense response to bacterium and the innate immune response
chr15	42380725	42380943	Hypo	−0.3037	6	ADM	Promoter	Involved in signal transduction
chr18	46457591	46457619	Hypo	−0.3654	3	TMEM149; IGFLR1	Promoter	Involved in protein binding activities
chrX	52808025	52808053	Hypo	−0.4230	5	MCART6; SLC25A53	Promoter	Not available
**Ileum lymph node**								
chr1	97612779	97612897	Hyper	0.4640	9	TERC; TR	5′ UTR	Involved in g-protein coupled receptor activity, response to stimulus and signalling
chrX	18020880	18020987	Hyper	0.4435	10	CCDC160	5′ UTR	Not available
chrX	79493022	79493221	Hyper	0.37542	8	NLGN3	5′ UTR	Roles in biological adhesion/regulation, cell adhesion, developmental process and signalling
chr1	66517241	66517269	Hyper	0.51326	3	ILDR1	Promoter	Roles in high-density lipoprotein particle receptor activity, protein binding; cellular response to leukemia inhibitory factor, endocytosis and positive regulation of peptide hormone secretion
chr3	20725467	20725535	Hyper	0.3824	5	HIST2H2AC; H2AC20	Promoter	Roles in cellular and metabolic processes
chr3	52530399	52530544	Hyper	0.2650	10	BARHL2	Promoter	Roles in cellular and metabolic processes
chr5	49488200	49488264	Hyper	0.3872	5	C5H12orf66	Promoter	Involved in cellular response to amino acid and glucose starvation, negative regulation of TORC1 signaling and protein localization to lysosome
chr5	50362472	50362729	Hyper	0.3306	8	AVPR1A	Promoter	Roles in response to stimulus, signalling and cellular processes
chr6	25666563	25666667	Hyper	0.3021	5	EIF4E	Promoter	Implicated in binding activities: DNA-binding, transcription factor binding, RNA 7-methylguanosine cap binding, RNA binding. Role in translation initiation factor activityetc.
chr6	65573034	65573154	Hyper	0.3145	6	GABRA4	Promoter	Roles in cellular processes, response to stimulus and signalling
chr7	25117101	25117262	Hyper	0.2676	7	SLC27A6	Promoter	Implicated in arachidonate-CoA ligase activity, long-chain fatty acid transporter activity and long-chain fatty acid-CoA ligase activityetc.
chr7	43711718	43711816	Hyper	0.5535	4	FAM174C; C7H19orf24	Promoter	Not available
chr7	83617422	83617724	Hyper	0.2904	10	HAPLN1; LP; CRTL1	Promoter	Implicated in extracellular matrix structural constituent conferring compression resistance, hyaluronic acid binding, cell adhesion, central nervous system development, extracellular matrix organization and skeletal system development
chr8	6824520	6824567	Hyper	0.2727	9	HPGD; PGDH	Promoter	Roles in cellular and metabolic processes
chr9	81419168	81419344	Hyper	0.3991	10	PLAGL1; ZAC1	Promoter	Implicated in DNA binding, DNA-binding transcription activator activity and metal ion bindingetc.
chr15	29699604	29699770	Hyper	0.3465	8	MIZF; HINFP	Promoter	Implicated in DNA binding, DNA-binding transcription factor activity and chromatin bindingetc.
chr22	52872082	52872205	Hyper	0.4424	8	TDGF1	Promoter	Roles in cellular and developmental processes, response to stimulus and signalling
chr25	2375675	2375804	Hyper	0.3033	6	PAQR4	Promoter	Implicated in signaling receptor activity
chrX	94769374	94769602	Hyper	0.2681	8	MSN	Promoter	Implicated in actin and cell adhesion molecule binding, T cell aggregation, cytoskeleton organization and establishment of epithelial cell apical/basal polarityetc.
chr2	124527051	124527172	Hypo	−0.3181	6	EPB41	5′ UTR	Roles in 1-phosphatidylinositol binding, phosphoprotein binding and protein-containing complex assemblyetc.
chr4	105525926	105525975	Hypo	−0.2783	4	LOC780933	5′ UTR	Involved in endopeptidase activity, metal ion binding, digestion, and proteolysissetc.
chr16	12701415	12701500	Hypo	−0.3002	4	RGS1	5′ UTR	Involved in G-protein alpha-subunit binding, GTPase activator activity, calmodulin binding and the immune response
chr1	96451609	96451735	Hypo	−0.4015	6	SLC2A2	Promoter	Implicated in D-glucose transmembrane transporter activity, fructose transmembrane transporter activity and in carbohydrate metabolic processetc.
chr1	115883148	115883250	Hypo	−0.3184	5	bta-mir-1246; MIR1246	Promoter	Not available
chr2	91231696	91231897	Hypo	−0.259	6	WDR12	Promoter	Implicated in protein binding, ribonucleoprotein complex binding and Notch signaling pathwayetc.
chr3	33247259	33247316	Hypo	−0.4231	3	MIR2285AS-3	Promoter	Not available
chr4	44625402	44625552	Hypo	−0.4710	3	SLC26A5	Promoter	Roles in anion:anion antiporter activity, bicarbonate transmembrane transporter activity and bicarbonate transportetc.
chr4	105504452	105504613	Hypo	−0.3705	5	TRYX3; PRSS58	Promoter	Involved in serine-type endopeptidase activity and proteolysis
chr4	105804315	105804451	Hypo	−0.3223	6	LOC509513; TCRB	Promoter	Involved in signaling receptor activity, T cell mediated cytotoxicity directed against tumor cell target and in detection of tumor cell
chr5	25685792	25685855	Hypo	−0.3334	3	ZNF385A	Promoter	Implicated in DNA binding, mRNA 3′-UTR binding and RNA bindingetc.
chr5	30081038	30081161	Hypo	−0.3327	4	NCKAP5L	Promoter	Role in cellular processes
chr5	61699340	61699492	Hypo	−0.2965	3	bta-mir-1251; MIR1251	Promoter	Role in gene silencing
chr6	89733241	89733396	Hypo	−0.2832	3	BTC	Promoter	Roles in cellular and metabolic processes, response to stimulus and signalling
chr6	116834070	116834098	Hypo	−0.3655	3	TMEM129	Promoter	Implicated in metal ion binding, ubiquitin protein ligase activity and protein ubiquitination
chr7	57416113	57416446	Hypo	−0.3427	10	RBM27	Promoter	Role in cellular process
chr8	7876115	7876240	Hypo	−0.3657	4	BLK	Promoter	Implicated in immune regulation, biological adhesion, biological regulation, cellular process, developmental process, metabolic process, response to stimulus and signalling
chr8	67283316	67283560	Hypo	−0.3761	4	SLC18A1	Promoter	Implicated in cellular process and secondary carrier transporter activities
chr8	74789324	74789518	Hypo	−0.2649	5	DNAJA1	Promoter	Involved in ATP binding, DNA damage response, detection of DNA damage and toxin transportetc.
chr8	76081883	76081965	Hypo	−0.3222	3	CCL21	Promoter	Roles in immune regulation, biological regulation, cellular process, metabolic process, response to stimulus and signalling
chr8	94567765	94567917	Hypo	−0.2923	8	NIPSNAP3A	Promoter	Involved in protein binding
chr18	51564992	51564998	Hypo	−0.3296	3	TEX101	Promoter	Roles in cellular processes
chr23	28335000	28335166	Hypo	−0.4334	4	DHX16	Promoter	Implicated in ATP binding, RNA binding and RNA splicingetc.
chr28	26458052	26458244	Hypo	−0.3898	7	PP; PPA1	Promoter	Implicated in cellular and metabolic processes
chrX	86776889	86777091	Hypo	−0.3039	9	SUV39H1	Promoter	Implicated in cellular and metabolic processes

a Chr, chromosome.

b MethyStatus, methylation status, hyper = hyper methylated, hypo = hypomethylated.

c MethDiff, methylation difference between infected and non-infected tissues.

d #CG, number of CpG sites in each differentially methylated region.

In the ILLN, the significant DMCs were annotated to 1717 genes including in the promoters or 5′UTRs of 119 genes (Supplementary Table S2D). The most significant DMCs were found in the introns of *SOBP* (5 DMC sites, FDR from 1.33E-05 to 3.67E-05), *PDE5A* (8 DMC sites, FDR from 1.92E-05 to 0.0083), *PRDM5* (2 DMC sites, FDR = 1.54E-04), *ZNF553*, (14 DMC sites in downstream region, FDR from 2.41E-04 to 0.0091), *UBAC2* (4 DMC sites, FDR from 8.43E-04 to 0.0018), *RORB* (16 DMC sites, FDR from 0.0022 to 0.0049) and promoter of *RBM27* (9 DMC sites, FDR from 5.03E-04 to 0.0064). A total of 422 genes harbored 6 to 64 DMCs including *NLGN1* (64 DMC sites in intron), bta-mir-2887–1 (57 sites in downstream region), PDE5A (52 sites in intron), *PTPRK* (38 sites in intron), *TENM1* (37 sites in intron) and *EFNAS* (36 sites in 3′UTR and intron), etc. Moreover, about 36 genes harbored 6 to 21 DMC sites in their promoters including miR-387–1 (21 sites), *SUV39H1* (18 sites), *MSN* (17 sites), *OTUD5* (17 sites), *NR2E1* (16 sites), *TMEM15* (13 sites), etc.

### Differentially Methylated Regions Between MAP-Infected and MAP-Negative Samples, and Distribution Patterns

**FIGURE 4:**
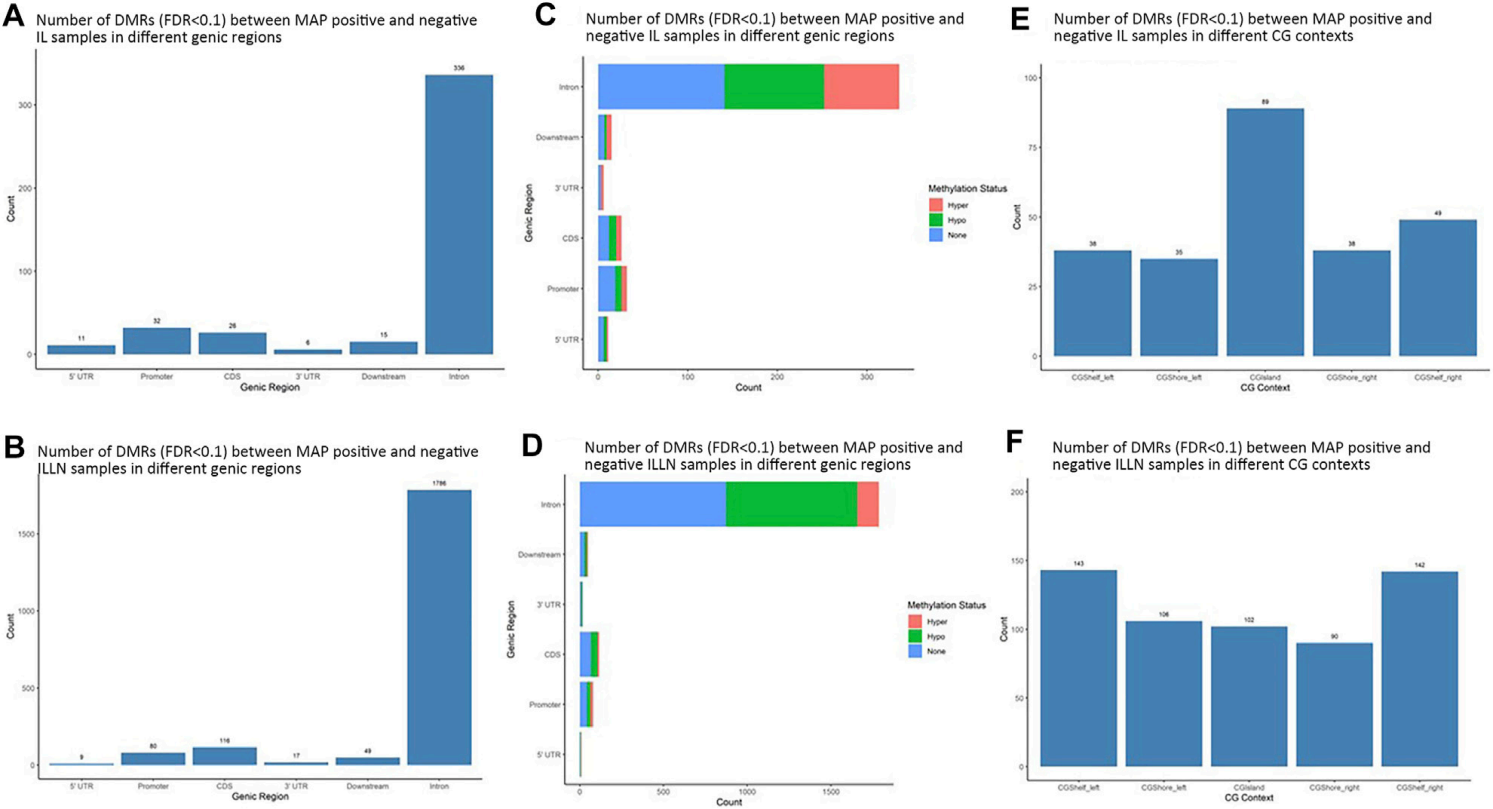
Distribution of significantly differentially methylated regions (DMR) in different genic regions between MAP-infected (MAPinf) and MAP-negative (MAPneg) cows in **(A)** ileum (IL) and **(B)** IL lymph node (ILLN), and corresponding methylation status in **(D)** IL and **(C)** ILLN. A DMR was considered hypermethylated if it had a positve methylation difference ≥25% between MAPinf and MAPneg samples. Similarly, a DMR was hypomethylated if it had a decreased methylation difference ≥25% between MAPinf and MAPneg samples. A DMR methylation status of none implies that the methylation difference was less than 25% in both scenarios. Distribution of differentially methylated cytosines in CpG island context in **(E)** IL and **(F)** ILLN samples between MAPinf and MAPneg samples.

Significant DMRs were 1259 and 8086 (FDR<0.1) between MAPinf and MAPneg IL and ILLN samples, respectively (Supplementary Tables S4A–D). Chromosomal distribution indicated a higher number of DMRs (≥60 DMRs) on BTA 1, 2, 3, 4, 5, 6, 7, 8 and 9 in IL and on (≥400 DMRs) BTA 1, 2, 3, 4, 5, 6, 7, 8, 9 and X in ILLN while the least numbers were found on BTA 22 (n = 8), 28 (n = 12) and 29 (n = 11) in the IL and on BTA 25 (*n* = 47), 28 (*n* = 57) and 29 (n = 69) in the ILLN (Supplementary Table S2E). Out of the total number of DMRs, 426 (IL) and 2057 (ILLN) were found in genic regions (Supplementary Tables S4B,D). Distribution of DMRs in different genic regions indicated highest proportions in intronic regions and the least numbers in 5′UTR and 3′UTR in both issues (Figures 4A,B). Similarly, more hypermethylated and hypomethylated DMRs were found in the introns and least numbers in 5′UTR and 3′UTR in both issues (Figures 4C,D).

Distribution of DMRs in CGI context indicated a total number of 249 and 583 DMRs in the IL and ILLN, respectively (Supplementary Tables S5A–B, Figures 4E,F). Out of these, 89 and 102 DMRs were located within CGIs. Relative to these CGIs, 73 and 87 were located in CG shelf left and right, respectively in IL, while more DMRs (249 and 232) were found in CGI shelf left and right, respectively, in ILLN (Figures 4E,F).

**FIGURE 5:**
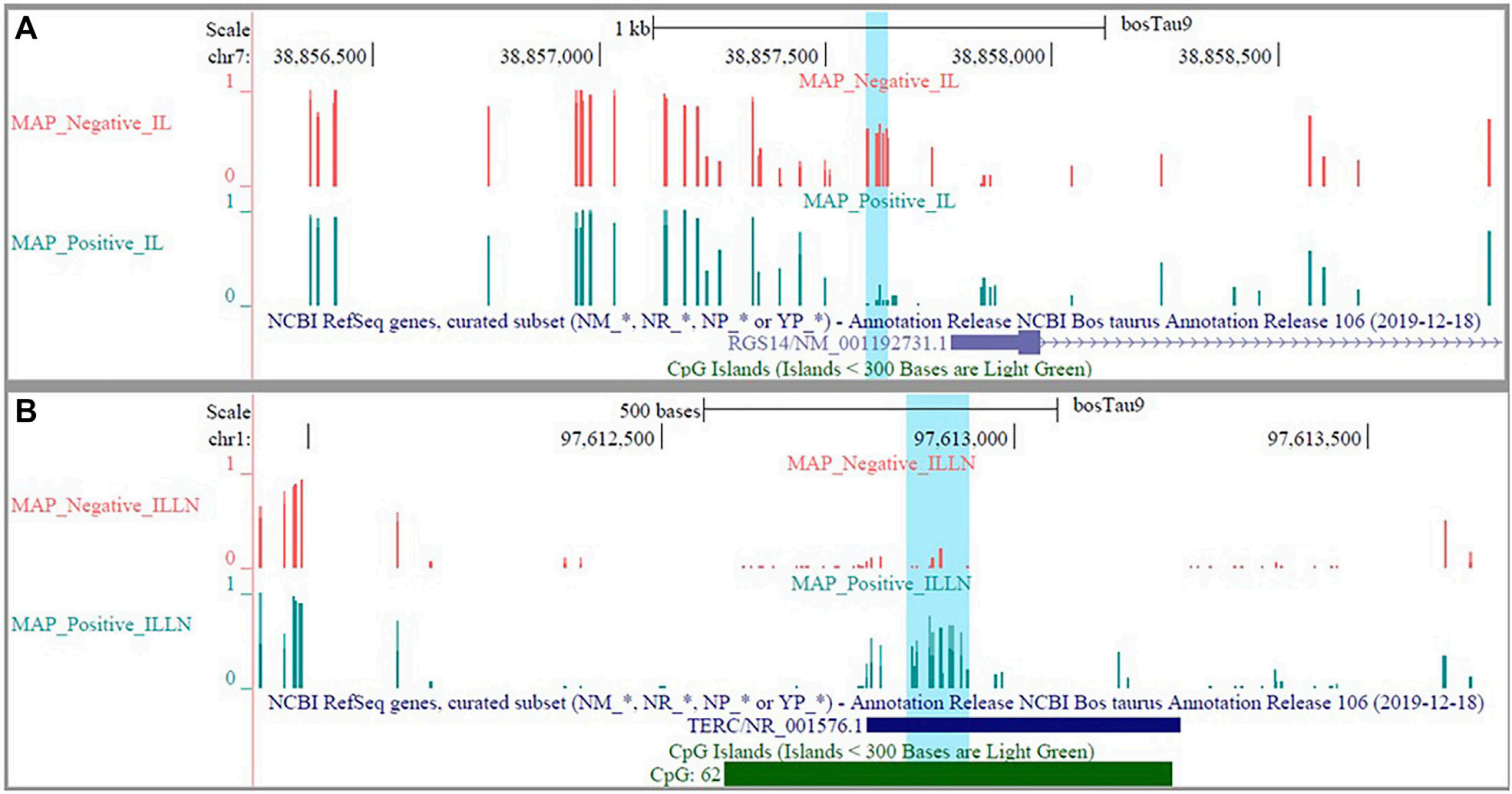
Browser shots showing **(A)** a hypomethylated DMR in a CpG island in *RGS14* gene in IL MAP-infected compared with negative samples and **(B)** a hypermethylated DMR in a CpG island in *TERC* gene in ILLN MAP-infected compared with negative samples.

The DMRs were annotated to 394 genes (here referred to as DMR genes) in the IL and to 1305 genes in the ILLN (Supplementary Tables S4B,D). Out of these, promoter and 5′UTR region DMRs were annotated to 43 genes in the IL including eight genes (*C1H21orf62, GST O 2, MAP6D1, EZH2, HSD17B11*, *bta-mir-181d, CCIN* and*, CCDC56*) with hypermethylated promoters and or 5′UTRs and 10 genes (*UBE2D2, ITGB1BP1, GLA, CHRNG, RGS14, CALM2, DEFB123, ADM, TMEM149, MCART6, SLC25A53*) with hypomethylated promoters or 5′UTRs (Supplementary Table S4B and Table 1). In the ILLN, DMRs were annotated to the promoters of 88 genes including, 19 genes with hypermethylated promoters and or 5′UTRs and 25 genes with hypomethylated promoters or 5′UTRs (Supplementary Table S4D and Table 1). Notable examples of genes with hypermethylated promoters or 5′UTRs included *TERC, EIF4E, PLAGL1*, CCDC160, ILDR1, HIST2H2AC, C5H12orf66, *FAM174C, TDGF1* etc., and with hypomethylated promoters or 5′UTRs included *SLC18A1, bta-mir-2285as-3, SLC2A2, SLC26A5, TRYX3, TMEM129, BLK, SLC18A1, CCL21, DHX16, PPA1,* etc. (Table 1). Browser screen shots of hypomethylated (*RGS14*) and hypermethylated (*TERC*) regions in two genes in the ILLN of MAPinf tissues are shown in Figure 5.

In order to understand the potential effects of methylation on the genes harboring DMRs, we examined their expression patterns in RNA-Seq data of the same samples (Ibeagha-Awemu et al., 2021). We found that, out of 394 and 1305 DMR genes in the IL and ILLN, respectively, 22 and 26, respectively, were also differentially expressed (FDR<0.1) at the mRNA level (Table 2).

**TABLE 2:** Select genes harboring differentially methylated regions and their gene (mRNA) expression patterns in the ileum and ileum lymph node of MAP-infected (MAPinf) compared to uninfected (MAPneg) cows.

		Differentially methylated region genes					mRNA expression^a^			
^b^Chr	Gene symbol	^c^Met status	^d^#CpGs	^e^#DMRs	Genic region	Gene	Base mean	L2FC^f^	p-value	Padj^g^
**Ileum**										
chr21	AMN	Hyper	3	1	Intron	AMN	710.6131	−1.5885	1.50E-07	4.99E-05
chr26	CPXM2	Hyper, hypo	8	2	Intron	CPXM2	130.4401	−2.3105	1.05E-06	0.0002
chr23	DEF6	Hypo	5	1	Intron	DEF6	208.6724	0.7429	0.0052	0.0970
chr3	GBP4	+	3	1	Intron	GBP4	994.8017	3.3463	0.0003	0.0139
chr8	HABP4	Hypo, −	6	2	Intron	HABP4	460.2397	−1.0853	0.0002	0.0097
chr4	IKZF1	+	3	1	Intron	IKZF1	436.6308	1.0129	0.0017	0.0469
chr15	INSC	+	3	1	Intron	INSC	30.7375	-1.5367	0.0005	0.0209
chr12	LCP1	+	6	1	Intron	LCP1	2878.195	1.5412	0.0007	0.0248
chr19	MGC137055	−	7	1	Promoter	MGC127055	951.7676	−1.3554	0.0003	0.0149
chr28	MTR	−	4	1	Intron	MTR	441.8496	−0.6786	0.0026	0.0634
chr18	PEPD; prolidase	Hypo	4	1	Intron	PEPD	1994.9530	−0.8194	0.0008	0.0276
chr6	PITX2	Hypo	4	1	Intron	PITX2	29.8845	−2.5165	0.0003	0.0138
chr6	PLAC8; PLAC8B	Hypo	8	1	Intron	PLAC8	234.1588	2.2503	0.0001	0.0070
chr13	PTPN1	+	3	1	Intron	PTPN1	400.9077	0.8147	0.0002	0.0112
chr8	SLC1A1	+	5	1	Downstream	SLC1A1	2182.2000	−1.7007	0.0004	0.0176
chr5	STAB2; hare	−	6	1	Intron	STAB2	75.9034	3.5210	5.28E-05	0.0047
chr2	STAT1	Hypo, −	13	2	CDS, intron	STAT1	8439.9450	1.4026	5.18E-05	0.0046
chr13	STK4	−	6	1	Intron	STK4	634.3818	0.7495	0.0008	0.0283
chr12	TNLG7A; TNFSF13B	Hypo	4	1	Intron	TNLG7A	99.1448	1.9854	0.0003	0.0147
chr5	TTLL1	−	3	1	Intron	TTLL1	129.2584	1.1507	0.0013	0.0394
chr9	VNN2	+	4	1	Intron	VNN2	254.2766	3.6994	1.19E-05	0.0017
**Ileum lymph node**										
chr3	AK5	−	7	2	Intron	AK5	11.1283	−3.0134	0.0003	0.0438
chr4	AOAH	−	6	1	Intron	AOAH	1144.6000	1.1055	3.61E-05	0.0114
chr8	CCL21	Hypo	3	1	Promoter	CCL21	2962.0750	−1.8148	0.0006	0.0595
chr6	CD38	Hypo, −	4	1	Intron	CD38	1415.7860	0.5503	0.0002	0.0332
chr5	CNTN1	Hypo, −	11	3	CDS, Intron	CNTN1	101.7895	−1.7744	0.0001	0.0236
chr1	CXADR	Hypo	4	1	Intron	CXADR	260.4728	1.9229	5.35E-06	0.0031
chr8	DMRT1	None	4	1	Intron	DMBT1	2668.1770	1.2567	0.0004	0.0460
chr8	EBF2	Hyper, Hypo	10	2	Intron, Intron	EBF2	17.5589	−2.9054	0.0005	0.0545
chr15	ELMOD1	+	5	1	Promoter	ELMOD1	9.7424	−3.3565	8.91E-05	0.0209
chr9	FHL5	−	9	2	Intron	FHL5	16.8634	−2.2775	0.0003	0.0450
chr3	GADD45A	Hyper	4	1	CDS	GADD45A	268.8654	1.2800	6.32E-05	0.0170
chr2	GALNT3	−	9	1	CDS	GALNT3	306.6631	−1.1925	2.07E-05	0.0084
chr26	GFRA1	Hypo	6	2	Intron	GFRA1	229.3136	−1.3818	7.97E-05	0.0200
chr7	GNG7	Hypo	6	1	Intron	GNG7	370.6341	1.3462	3.23E-05	0.0105
chr8	GRIN3A	Hypo	6	1	Intron	GRIN3A	22.4595	−2.8343	0.0007	0.0664
chr6	LEF1	+	10	1	Promoter	LEF1	1718.1730	−1.3951	0.0010	0.0886
chr6	MTHFD2L	Hypo	5	1	Intron	MTHFD2L	61.8015	−1.5939	0.0003	0.0409
chr9	PRDM1	−	5	1	Promoter	PRDM1	1057.196	0.9168	2.70E-07	0.0003
chr22	PRSS45	−	5	1	Intron	PRSS45	17.6579	−2.4531	2.77E-05	0.0096
chr5	PTPRQ	Hypo, +, −	33	8	Downstream, Introns	PTPRO	79.2753	1.3915	1.92E-07	0.0002
chr8	SLC24A2	Hypo	9	3	Intron	SLC24A2	28.5550	−1.563	0.0012	0.0916
chr3	SPTA1	+	6	1	Intron	SPTA1	27.9195	−3.1868	0.0002	0.0336
chr2	STAT4	−	3	1	Intron	STAT4	449.2908	−1.1274	0.0011	0.0904
chr9	STXBP5	Hypo	3	1	Intron	STXBP5	852.2494	−0.6002	0.0006	0.0628
chr9	THEMIS	Hypo, -	8	2	CDS, intron	THEMIS	975.8944	−1.3937	0.0001	0.0229
chr6	UGT8	Hypo	3	1	Intron	UGT8	77.1915	−2.1056	1.76E-06	0.0012

a mRNA, expression data is from Ibeagha-Awemu et al., 2021.

b Chr: chromosome.

c MethyStatus: methylation status, hyper = hyper methylated, hypo = hypomethylated, “+” = increased methylation level, “−” = decreased methylation level.

d #CpGs: number of CpG sites.

e #DMRs: number of differentially methylated regions.

f L2FC: Log2foldchange.

g Padj: p-values adjusted according to Benjaminin and Hochberg correction for false discovery rate.

### Functional Roles of Genes Harboring DMRs

**FIGURE 6:**
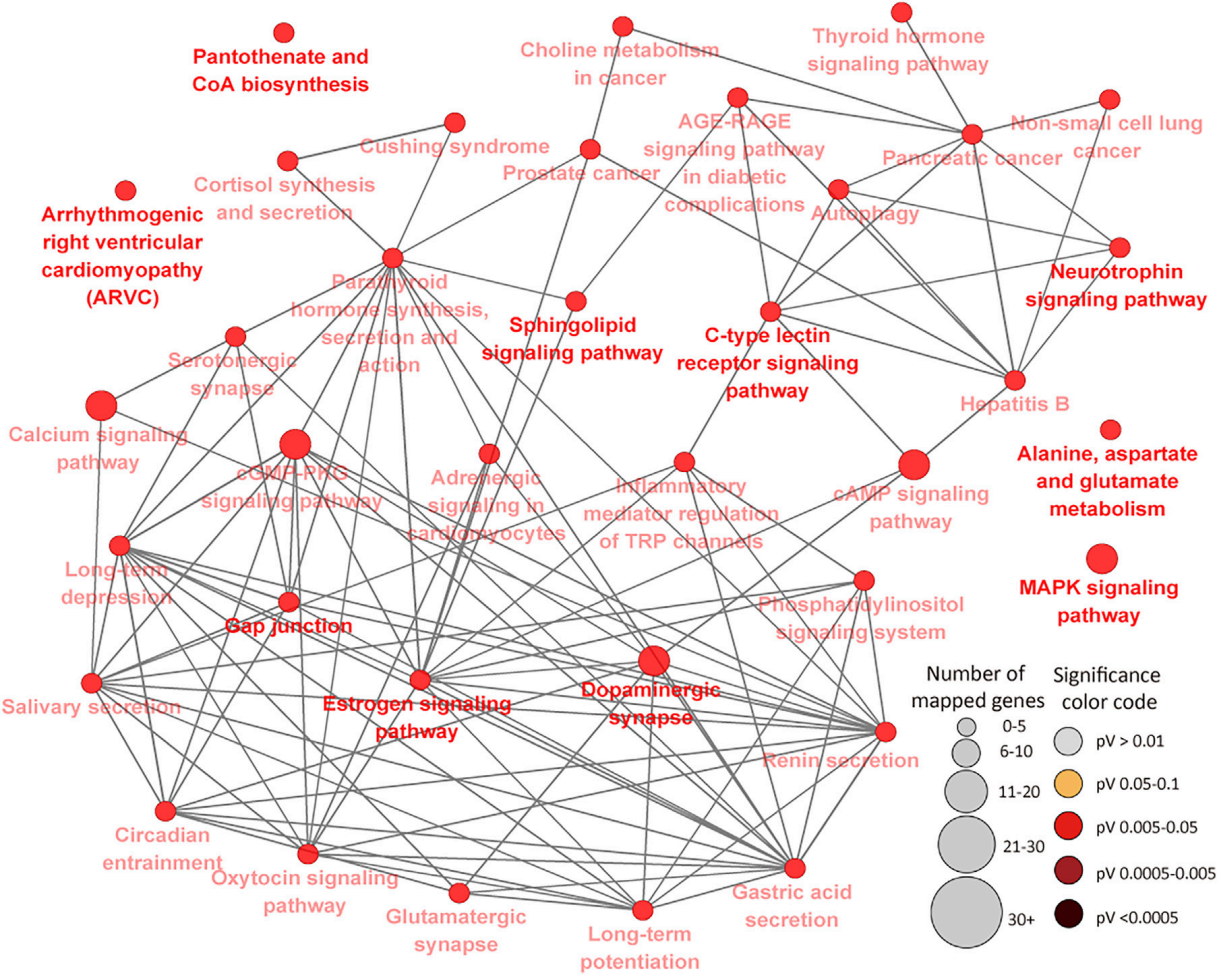
KEGG Pathways enriched for ileal tissue differentially methylated region genes showing connections between pathways. Each node represents a pathway and the size (the larger the node, the higher the number of enriched genes) and color (the deeper the color, the more significant the enriched pathway is) of the node represents the number of enriched genes and level of significance, respectively. Straight lines connect pathways.

To further determine the potential effects of cytosine methylation on the functions of genes, DMR genes were subjected to GO and pathways analysis. GO analysis of 394 DMR genes in the IL resulted in 75 BP, 11 MF and 19 CC GO terms (FDR<0.05) (Supplementary Tables S6A–C). Most of the enriched IL BP GO terms are related to cellular processes, metabolic processes, transport and system development while very few enriched terms (less than 1%) are related to disease and the immune process (Supplementary Table S6A). The most significant IL MF and CC GO terms were neurotransmitter receptor activity (FDR = 0.011) and synapse part (FDR = 0.006), respectively (Supplementary Tables S6B,C). A total of 36 KEGG pathways were enriched for IL DMR genes (Supplementary Table S6D) (Figure 6). The most enriched pathways included Gap junction (FDR = 0.009), Long-term depression (FDR = 0.009), Long-term potentiation (FDR = 0.011) and Sphingolipid signaling pathway (FDR = 0.011). Several disease and immune elated pathways were enriched for IL DMR genes, including C-type lectin receptor signaling pathway (FDR = 0.018), Inflammatory mediator regulation of TRP channels (FDR = 0.024), Cushing syndrome (FDR = 0.023); Hepatitis B (FDR = 0.037), Prostate cancer (FDR = 0.038), Non-small cell lung cancer (FDR = 0.037), Pancreatic cancer (FDR = 0.044), etc. Interestingly, some genes (n = 21) were enriched in many pathways, from 4 to 23 pathways (Supplementary Table S6E).

**FIGURE 7:**
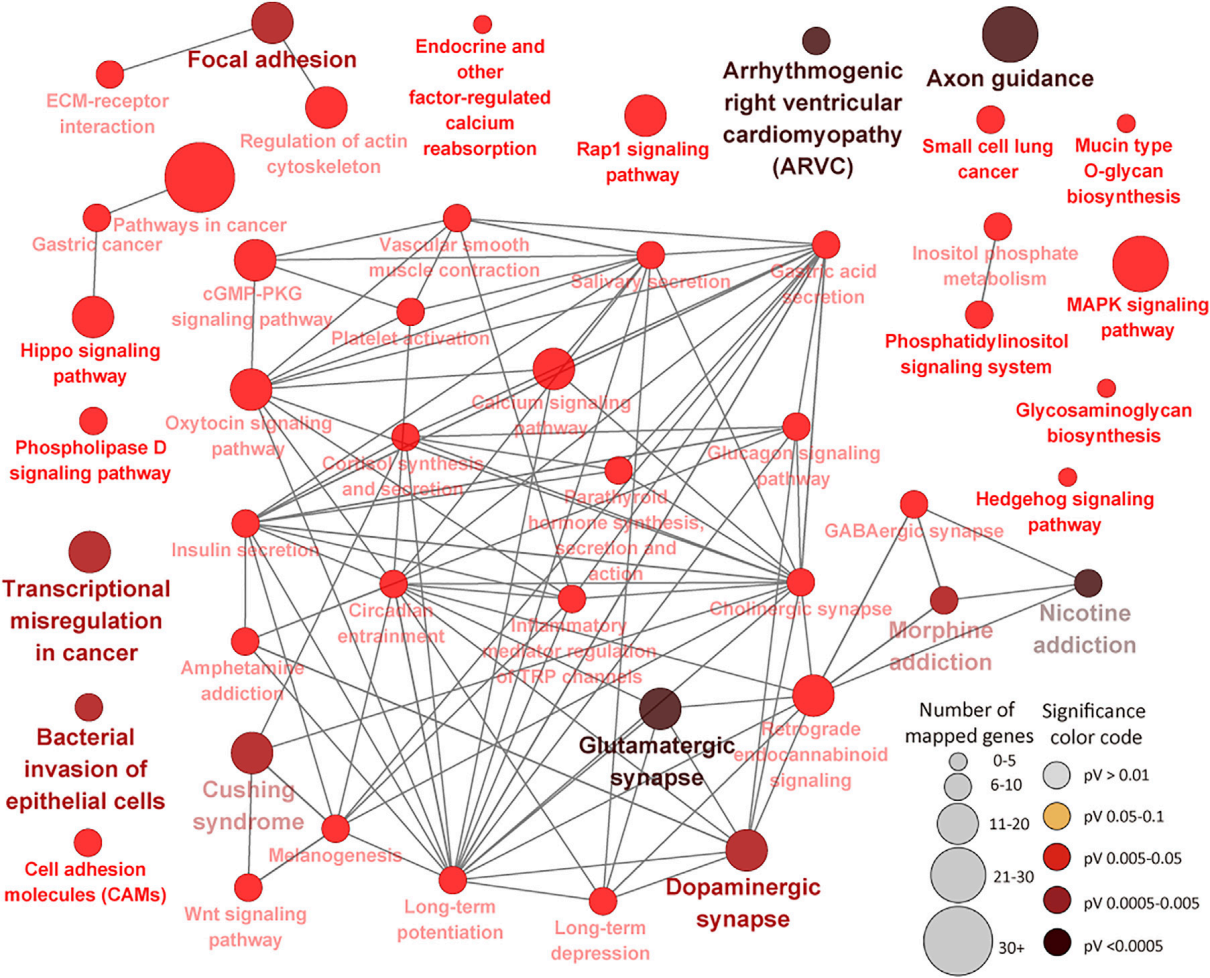
KEGG Pathways enriched for ileal lymph node differentially methylated region genes showing connections between pathways. Each node represents a pathway and the size of the node represents the number of enriched genes (the larger the node, the higher the number of enriched genes) while the color intensity represents the level of significance (the deeper the color, the more significant the enriched pathway is). Straight lines connect pathways.

The 1305 ILLN DMR genes were enriched for 374 BP, 91 MF and 89 CC GO terms (Supplementary Tables S7A–C). Most of the enriched BP terms are related to cellular processes (27.6%), biological regulation (16.6%), metabolic processes (15.4%) and response to stimulus (8.2%) (Supplementary Table S7A). More disease/immune related BP GO terms were enriched (FDR <0.05) for ILLN DMR genes as compared to IL DMR genes. Cytoskeletal protein binding (FDR = 6.94E-07) and plasma membrane part (FDR = 4.36E-12) were the most significantly enriched MF and CC GO terms, respectively (Supplementary Tables S7B,C). Forty-seven KEGG pathways were enriched for ILLN DMR genes including immune (e.g., Bacterial invasion of epithelial cells, Transcriptional misregulation in cancer, Cell adhesion molecules (CAMs), Inflammatory mediator regulation of TRP channels and Platelet activation, etc.) and disease (e.g., Cushing syndrome, pathways in cancer, Small cell lung cancer, Gastric cancer and Arrhythmogenic right ventricular cardiomyopathy (ARVC), etc.) related pathways (Figure 7; Supplementary Table S7D). Several metabolic pathways as well as cGMP-PKG signaling pathway were also enriched for ILLN DMR genes. A total of 63 genes were enriched in four to 28 pathways (Supplementary Table S7E).

A comparison of enriched pathways in both tissues indicated that 17 (FDR<0.05) pathways were commonly enriched for DMR genes in both tissues (SupplementaryTable S6F), including cGMP-PKG signaling pathway, MAPK signaling pathway, Oxytocin signaling pathway, Cushing syndrome, Arrhythmogenic right ventricular cardiomyopathy (ARVC) and Gastric acid secretion, etc. Furthermore, we compared the BP GO terms and pathways enriched for DMR genes with BP GO terms and pathways enriched for DE genes (Ibeagha-Awemu et al., 2021) in both tissues. Results indicated that 16 BP GO terms were commonly enriched for DMR genes and DE mRNAs in the IL, including regulation of cell migration, positive regulation of cell migration, negative regulation of growth, import into cell, positive regulation of B cell activation, regulation of mononuclear cell migration and lipid biosynthetic process, etc. (Supplementary Table S6G). Meanwhile, only C-type lectin receptor signaling pathway was commonly enriched for DMR genes and DE mRNAs in this tissue (IL) (Supplementary Table S6H). In the ILLN, only one BP GO term (regulation of B cell activation) was commonly enriched for DMR genes and DE genes (Supplementary Table S7F), while no KEGG pathway was commonly enriched.

## Discussion

Several studies have shown that MAP alters the expression of genes, biological processes and pathways following MAP infection or during JD progression (Shin M. K. et al., 2015; Hempel et al., 2016; Alonso-Hearn et al., 2019; Ariel et al., 2020; Facciuolo et al., 2020; Ibeagha-Awemu et al., 2021). In addition, a few studies have reported the involvement of miRNAs in JD (Farrell et al., 2015; Gupta et al., 2018; Wang et al., 2019a; Wang et al., 2019b). Our recent studies suggested a possible role of epigenetic processes in the mRNA transcriptome response of bovine monocyte derived macrophages infected by MAP (Ariel et al., 2020; Marete et al., 2021a). Moreover, we observed that MAP initiated a heightened immune response in the IL as compared to a likely suppressed immune response in the ILLN tissues of cows with subclinical MAP infection, which could be underpinned by epigenetic processes (Ibeagha-Awemu et al., 2021). Thus, this study investigated the occurrence of epigenetic regulation through whole genome DNA methylation status analysis of ileal and ileal lymph node tissues in response to persistent MAP-infection in dairy cows.

Overall, DNA methylation data showed differential tissue responses to subclinical MAP infection between ileal and ileal lymph node tissues. More significant DMCs, DMRs and CGIs were recorded between MAPinf and MAPneg cows in the ILLN as compared to the IL. This suggests that, the DNA methylation state of cells in the ILLN were altered to a greater extent by the presence of MAP pathogen than in the IL. This observation corroborates data on differentially expressed mRNA transcriptome of the same samples which suggested a potentially suppressed immune state in the ILLN as compared to a heightened immune state of the IL (Ibeagha-Awemu et al., 2021). It seems that DNA methylation profile is a signature that distinguishes MAPinf status from MAPneg status. It has been revealed that disease-specific DNA methylation profiles of intestinal epithelial cells from the ascending colon could accurately separate Inflammatory bowel disease patients from healthy controls with a sensitivity of 75% while ileal methylation signatures were capable of distinguishing Crohn’s disease from ulcerative colitis patients with a precision of 77% (Howell et al., 2018). Moreover, a participatory role of DNA methylation in the pathogenesis of MAP infection in cattle demonstrated by our data corroborates data from several studies showing aberrant changes of DNA methylation in Crohn’s disease (similar pathology as JD) and other Inflammatory bowel disease conditions (Karatzas et al., 2014; Somineni et al., 2019; Zeng et al., 2019; Li Yim et al., 2020).

Generally, only a small proportion of significant DMCs and DMRs were annotated to genic regions, 15.4 and 33.84% in IL and 12.3 and 25.44% in ILLN, respectively, indicating that majority of DMCs that may paly a role in JD are located outside of protein coding genes. This observation is in line with results of numerous genome wide association studies which have associated more variants located outside of protein coding genes with cattle production and disease traits (Jiang et al., 2010; Wang et al., 2012; Ibeagha-Awemu et al., 2016). The functional role of the DMCs and DMRs in the intergenic regions are not yet known but their impact could be potential action in *cis* or *trans* to genes. Generally, the methylation levels in the regulatory regions (promoter and 5′UTR) of genes were lower as compared to other genic regions in both tissues. This result substantiates previous findings with the method of WGBS in cattle (Zhou et al., 2018; Duan et al., 2019). Meanwhile, higher numbers of DMCs and DMRs were recorded in the intron regions as compared to other genic regions in both tissues. It is generally acknowledged that DNA methylation in promoter regions provides a mechanism of regulation of gene expression. More hypomethylated and hypermethylated DMRs were identified in the promoter/5′UTRs of genes in the ILLN as compared to the IL (Table 1), suggesting potential effects of DNA methylation on gene expression, especially in the ILLN. Majority of the identified genes with hypo/hypermethylated DMRs in their promoters or 5′UTRs have roles in multiple processes like cellular and/or metabolic processes, and immune regulation/response to stimuli (e.g., *BARHL2, BLK, BTC, CCL21, GLA, CHRNG,H2AC20, GABRA4*, *PPA1, SLC18A1*. *PLAGL1, EIF4E, TDGF1,* etc.). Some of these genes have been associated with mycobacterial infections including JD and other human diseases. For example, *BARHL2*, a member of BarH family of homeodomain proteins is a transcriptional regulator and also known to impact cell fate specification, cell differentiation, survival and migration (Juraver-Geslin et al., 2011; Ding et al., 2012). Moreover, DNA methylation of *BARHL2* in exosomal DNA from gastric juice has been recognized as a predictive biomarker of gastric cancer (Yamamoto et al., 2016). *BLK,* a non-receptor tyrosine kinase involved in cell proliferation and differentiation has roles in B-cell receptor signaling and B-cell development. *BLK* was among highly downregulated genes following MAP infection in mice (Shin M.-K. et al., 2015). *CCL21* is a chemokine which functions in recruiting T cells and has been associated with inflammatory bowel disease in human (Kinchen et al., 2018) and JD in cattle (Ibeagha-Awemu et al., 2021). *H2AC20* gene encodes a replication-dependent histone that is a member of the histone H2A family and is associated with Riddle Syndrome and Xeroderma Pigmentosum Group E disease. *PPA1* like other inorganic pyrophosphatases is important for phosphate metabolism in cells. *SLC18A1, SLC27A6, SLC2A2, SLC25A53 and SLC26A5,* members of the solute carrier family have varied functions including participation in fatty acid/cholesterol transport, regulation of lymphocyte signalling and modulation of diverse metabolic pathways (Song et al., 2020). Gene expression data of the same samples indicated that 36 members of the solute carrier gene family were significantly regulated by the presence of MAP (Ibeagha-Awemu et al., 2021). It is known that MAP and other mycobacteria acquires and utilises host-derived fatty acids and cholesterol for their sustenance (Wilburn et al., 2018). For example, *SCL27A6* encodes FATP6, a member of the fatty acid transport protein family known to mediate the uptake of long chain fatty acids (Stahl et al., 2001). A recent study demonstrated involvement of *SCL27A6* in fatty acid transport into the mammary gland and how its overexpression alters fatty acid metabolism in the bovine mammary gland (Zhang et al., 2021). Therefore, alteration of the function of SCL27A6 and probably the other solute carriers due to DNA methylation may have consequences on pathogen survival during MAP infection. EIF4E expression has been reported as changed by MAP in Holstein cattle (Ibeagha-Awemu et al., 2021). *PLAGL1* functions as a suppressor of cell growth and its promoter methylation or deletion has been reported in cancerous cells (Peille et al., 2013; Li et al., 2014).

Results of functional analysis of IL and ILLN DMR genes further portrayed the potent effect of MAP on DNA methylation in the ILLN tissue as compared to IL tissue, whereby more GO terms and KEGG pathways were enriched for ILLN DMR genes as compared to IL DMR genes. In the IL, majority of enriched BP GO terms are related to cellular processes, metabolic processes, transport and system development processes suggesting that these processes were more sensitive to DNA methylation as opposed to only about 1% of BP GO terms being disease/immune related (e.g. regulation of mononuclear cell migration and positive regulation of B cell activation). Meanwhile, more immune/disease pathways and BP GO terms were enriched for ILLN DMR genes, suggesting that the immune response was sensitive to regulation by DNA methylation in this tissue. This result corroborates our recent findings of an activated immune state in the IL and a depressed immune response in the ILLN in response to MAP presence (Ibeagha-Awemu et al., 2021).

In the IL, it was interesting to note that 22 genes were enriched for 4 or more pathways, including *PLCB1* (23 pathways), *GNAI1* (22 pathways), *ITPR1* (21 pathways), *RAF1* (20 pathways), *CALM2* (16 pathways), *PIK3R3* (15 pathways), *MAPK10* (12 pathways), *CREB5* (10 pathways) and *BCL2* (9 pathways), etc., indicative of potentially important regulatory effects of DNA methylation of these genes and corresponding pathways during MAP infection. This is supported by the fact that some of them (genes and pathways) have been associated with JD in cattle and human diseases (e.g. cancer, tuberculosis, leishmaniasis, etc). For example, *GNAI1* and *GNAI3* were found to reduce colitis-associated tumorigenesis in mice through blocking of IL6 signaling and down-regulation of the expression of *GNAI2* (Li et al., 2019). Moreover, *GNAI1* was found as enriched in four pathways in human macrophages following infection by Leishmania (Fernandes et al., 2016). *ITPR1* which regulates important molecules like platelets in the innate and adaptive immune system was recently found in a quantitative trait loci (QTL) region for JD in Jersey cattle (Kiser et al., 2017). Furthermore, many identified GWAS (genome wide association study) associated genes or genes within QTL regions for JD like *PPFIBP1, ERGIC2, ITPR2, LEF1, DKK2, DEF6, CCDC91,* etc., (Gao et al., 2018; Mallikarjunappa et al., 2018), presented different methylation status between MAPinf and MAPneg cows in this study, suggesting a possibility of mechanistic interactions between epigenetic and genetic signals in JD progression. *CALM2*, known to be involved in the regulation of a large number of enzymes and other proteins was reported as downregulated during MAP enteric colonization of ileal tissues of neonatal calves (Khare et al., 2012). Meanwhile, *CREB5* was among upregulated genes during tuberculosis infection in humans (Alam et al., 2019). Majority of the pathways enriched for IL DMR genes were highly interconnected (Figure 6) and notable ones like MARK Signalling pathway, C-type lectin receptor signaling pathway, Cushing syndrome, Inflammatory mediator regulation of TRP channels, cAMP signaling pathway have reported associations with JD and other mycobacterial infections (Rybaczyk et al., 2009; Ariel et al., 2020; Ibeagha-Awemu et al., 2021) or are being associated with JD for the first time.

Enrichment of more immune/disease related pathways for ILLN DMR genes as compared to IL DMR genes including notable pathways like Bacterial invasion of epithelial cells, Cushing syndrome, Transcriptional misregulation in cancer, Pathways in cancer, Inflammatory mediator regulation of TRP channels, Cell adhesion molecules (CAMs), Platelet activation, amongst others, emphases the involvement of DNA methylation in the immune response processes to MAP presence in this tissue. Many ILLN DMR genes (*n* = 63) were enriched in four to 28 pathways suggesting central roles in JD. For example, the top ILLN pathways driving genes like *CACNA1A* (10 pathways), *CAMK2A* (14 pathways), *CAMK2D* (16 pathways), *CREB3L2* (10 pathways), *GNAQ* (24 pathways), *GSK3B* (10 pathways), *ITPR2* (19 pathways), *PLCB1* (28 pathways), *PPP3CA* (10 pathways), *PRKACB* (26 pathways), *PRKCB* (27 pathways), *ADCY1* (22 pathways), etc., are mainly involved in binding, catalytic and transporter activities. Apart from a reported role of *GSK3B* in the regulation of milk synthesis and proliferation *via* mTOR/S6K1 signaling pathway in dairy cow mammary gland epithelial cells (Zhang et al., 2014) or report that MAP fibronectin attachment protein (FAP) mediated dendritic cell CD8^+^ T cell proliferation and cytotoxic T lymphocyte activity is *via GSK-3* (Noh et al., 2012), there are no reports of association of these genes with bovine JD. Considering that these genes are enriched in an appreciable number of immune/disease pathways during subclinical MAP infection, their roles in JD pathogenesis is worth investigating.

The Bacterial invasion of epithelial cells pathway (FDR = 0.002) and Focal adhesion pathway (FDR = 0.002) were among the top enriched pathways for ILLN DMR genes. The Focal adhesion pathway plays crucial roles in key biological processes such as cell motility, cell proliferation, cell differentiation, regulation of gene expression and cell survival, etc. Signalling events initiated by cell-matrix adhesions results in morphological alterations and modulation of gene expression. Activated focal adhesion kinase complexes with Src family kinases and other proteins to initiate multiple downstream signaling pathways to regulate diverse cellular functions, and are implicated in various human diseases including cancers (Zhao and Guan, 2011). Cell adhesion and Cell communication pathways have been linked to paratuberculosis in sheep (Gossner et al., 2017) and inflammatory bowel diseases (ulcerative and Crohn’s disease) in humans and model animals (Ma et al., 2010; Brooks et al., 2017). Emergence of the Bacterial invasion of epithelial cells pathway as a top pathway in this study is not surprising knowing that MAP through various mechanisms internalizes in sub-epithelial macrophages where it effectively subverts host cell responses to promote intracellular survival and replication, and DNA methylation may be one of the regulating mechanisms. Although several pathways were commonly enriched in both tissues (e.g. cGMP-PKG signaling pathway, Cushing syndrome, Inflammatory mediator regulation of TRP channels, MAPK signaling pathway, Gastric acid secretion pathway, etc.), the majority of the implicated DMR genes where different between the two tissues (Supplementary Tables S6D,F, S7D), again attesting to the differential regulation of the two tissues by DNA methylation alteration.

Interestingly, comparison of the present data with mRNA-Seq data (Ibeagha-Awemu et al., 2021) of the same samples indicated that mostly BP GO terms in the IL (n = 16) were commonly enriched for IL DMR genes (this data) and genes expressed (mRNA-seq) in the IL tissues. Most of the terms are implicated in the movement of cells. Meanwhile in the ILLN only regulation of B cell activation BP GO term was commonly enriched for DMR genes and DE genes, while no KEGG pathway was commonly enriched. This is not surprising given that only a limited number of BP GO terms (*n* = 10) and KEGG pathways (*n* = 7.) were enriched for differentially expressed genes in this tissue (ILLN) (Ibeagha-Awemu et al., 2021). Given the high number of DMCs and DMRs identified in the ILLN and the lower number of DE genes identified in the same samples (Ibeagha-Awemu et al., 2021), it can be deduced that, the DNA methylation state of the cells, repressed the host cellular immune response to the presence of MAP, and therefore suggest a role for DNA methylation in the regulation of the host response to MAP infection. Moreover, comparison of the DNA methylation data with gene expression data of the same animals (Table 2) suggested that DNA methylation in other gene regions also potentially affected the expression of the corresponding genes. For example, the promoter methylation levels of three genes (*ELMOD1, LEF1* and *PRDM1*) and intron region methylation levels of 12 genes (*AMN, DEF6, PLAC8, STAB2, STAT1, STK4, TNFSF13B, AOAH, CD38, CXADR, GNG7* and *SPTA1*) were inversely related to gene expression. In most cases however, the state of DNA methylation was not inversely related to gene expression. This data suggests that JD development may be associated with anomalous methylation.

## Conclusion

This data is the first to demonstrate a participatory role of DNA methylation in the pathogenesis of MAP infection in dairy cattle. DNA methylation analysis demonstrated differential tissue responses to MAP infection with more DMCs and DMRs identified in ILLN tissues as compared to IL tissues of MAPinf compared to MAPneg cows, indicating that the ILLN was more impacted by regulation by DNA methylation as compared to IL tissue. Moreover, immune processes in the ILLN were more impacted by DNA methylation compared to the IL. Genes with hypermethylated (e.g. *GSTO2, CCDC56, TERC, NLGN3, H2AC20, BARHL2, AVPR1A, EIF4E, GABRA4, PGDH, etc.)* or hypomethylated (e.g. *UBE2D2, ITGB1BP1, GLA, CHRNG, NCKAP5L, BTC, RBM27, BLK, SLC18A1, CCL21, TEX101, PPA1 and SUV39H1, etc.)* promoters as well as pathways driving DMR genes (e.g. *CACNA1A*, *CAMK2A*, *CAMK2D*, *CREB3L2*, *GNAQ, GSK3B*, *ITPR2*, *PLCB1*, *PPP3CA*, *PRKACB*, *PRKCB*, *ADCY1, etc.)* identified between MAPinf and MAPneg cows in this study could be potential biomarkers of MAP infection, which can support development of improved diagnostic and therapeutic solutions for JD management.

## Data Availability

The datasets presented in this study can be found in online repositories and in the article/Supplementary Material. The raw sequence reads generated for this study have been deposited in the NCBI Sequence Read Archive (SRA) under the BioProject ID PRJNA771921.
