# Supplementary figures and images for: Whole Genome Methylation Analysis Reveals Role of DNA Methylation in Cow’s Ileal and Ileal Lymph Node Responses to Mycobacterium avium subsp. paratuberculosis Infection
Source: Front Genet. 2021 Dec 21;12:797490. doi: 10.3389/fgene.2021.797490 (PMC8724574; doi:10.3389/fgene.2021.797490)

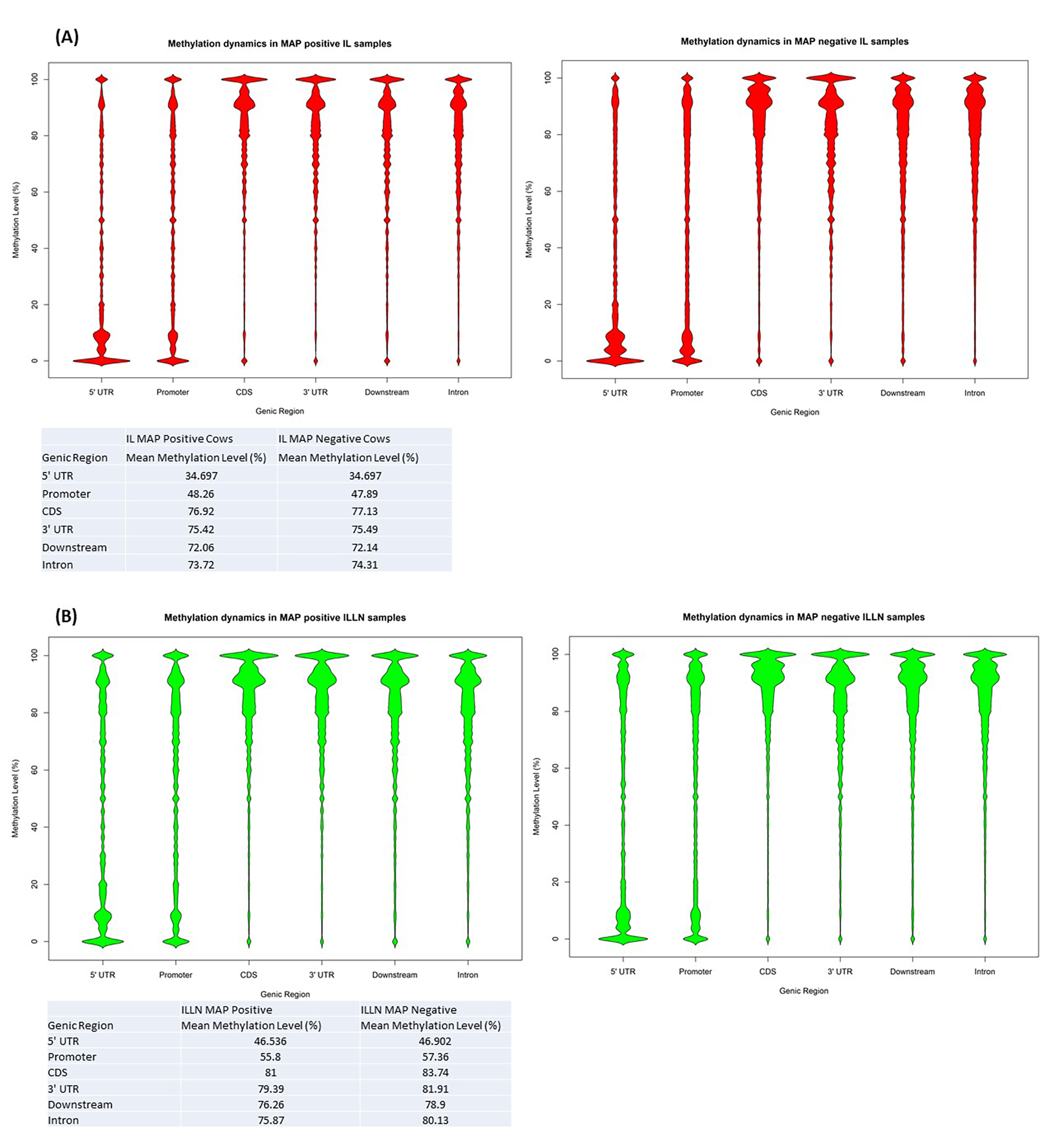

Supplement: Supplementary file 3 [file Image3.TIF]

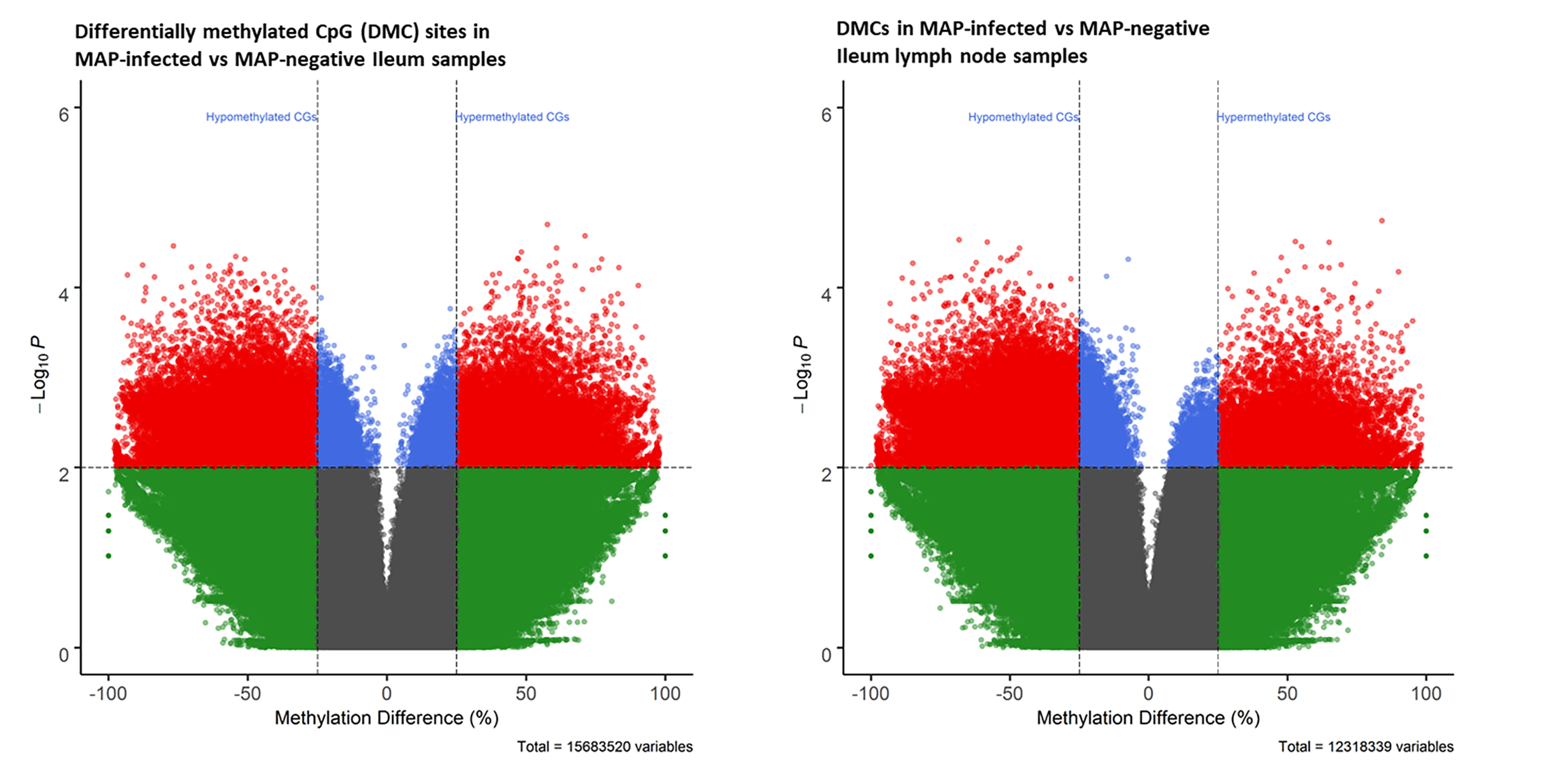

Supplement: Supplementary file 4 [file Image4.TIF]

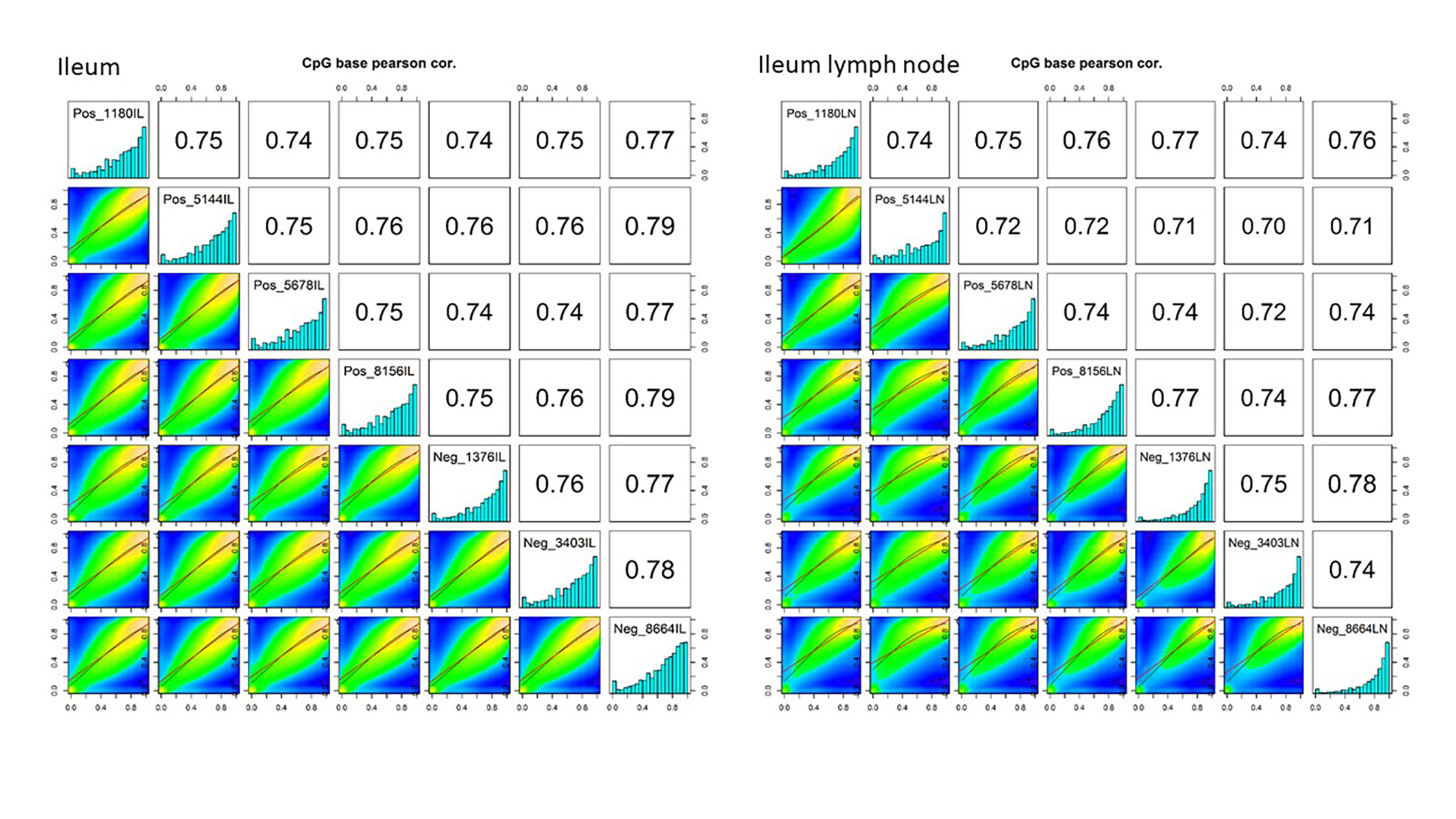

Supplement: Supplementary file 5 [file Image1.JPEG]

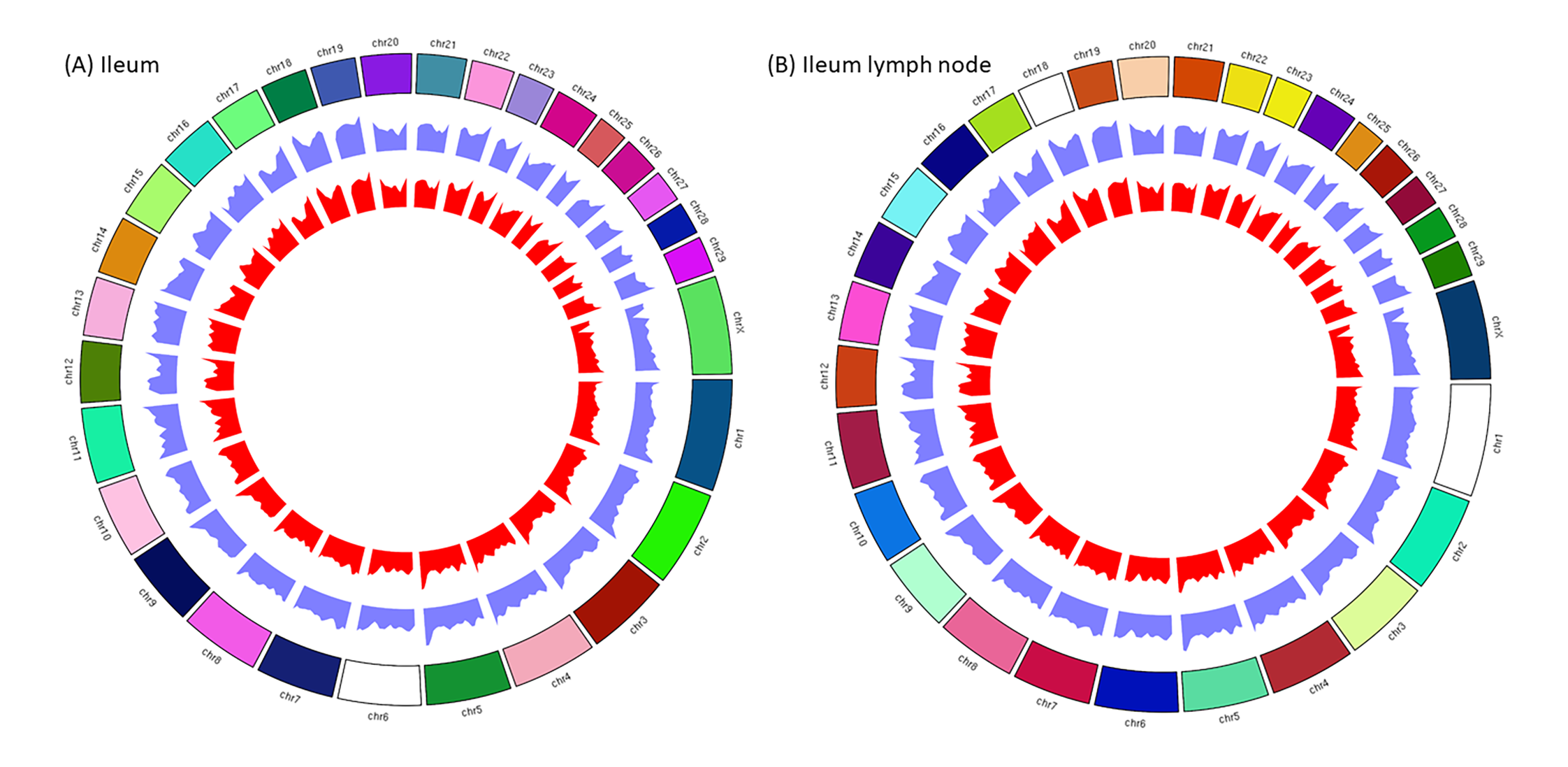

Supplement: Supplementary file 6 [file Image2.TIF]
